# Customized Scaffolds for Direct Assembly of Functionalized DNA Origami

**DOI:** 10.1021/acsami.3c05690

**Published:** 2023-06-02

**Authors:** Esra Oktay, Joshua Bush, Merlyn Vargas, Dylan Valerio Scarton, Bailey O’Shea, Amber Hartman, Christopher M. Green, Kayla Neyra, Carolina M. Gomes, Igor L. Medintz, Divita Mathur, Remi Veneziano

**Affiliations:** † College of Engineering and Computing, Department of Bioengineering, George Mason University, Manassas, Virginia 20110-2201, United States; ‡ College of Science, Interdisciplinary Program in Neuroscience, George Mason University, Fairfax, Virginia 22030-4444, United States; § Institute for Advanced Biomedical Research, Manassas, Virginia 20110-2201, United States; ∥ Center for Bio/Molecular Science and Engineering Code 6900, U.S. Naval Research Laboratory, Washington DC 20375-0001, United States; ⊥ Department of Chemistry, Case Western Reserve University, Cleveland, Ohio 44106-7078, United States

**Keywords:** single-stranded DNA, DNA origami, scaffold, asymmetric polymerase chain reaction, bioconjugation, DNA nanotechnology

## Abstract

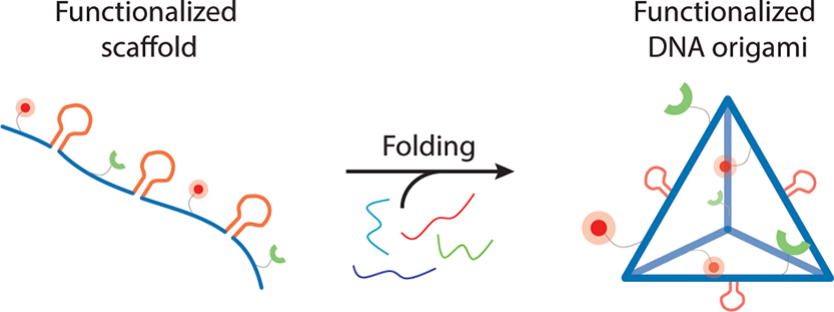

Functional DNA origami nanoparticles (DNA-NPs) are used as nanocarriers in a variety of biomedical applications including targeted drug delivery and vaccine development. DNA-NPs can be designed into a broad range of nanoarchitectures in one, two, and three dimensions with high structural fidelity. Moreover, the addressability of the DNA-NPs enables the precise organization of functional moieties, which improves targeting, actuation, and stability. DNA-NPs are usually functionalized via chemically modified staple strands, which can be further conjugated with additional polymers and proteins for the intended application. Although this method of functionalization is extremely efficient to control the stoichiometry and organization of functional moieties, fewer than half of the permissible sites are accessible through staple modifications. In addition, DNA-NP functionalization rapidly becomes expensive when a high number of functionalizations such as fluorophores for tracking and chemical modifications for stability that do not require spatially precise organization are used. To facilitate the synthesis of functional DNA-NPs, we propose a simple and robust strategy based on an asymmetric polymerase chain reaction (aPCR) protocol that allows direct synthesis of custom-length scaffolds that can be randomly modified and/or precisely modified via sequence design. We demonstrated the potential of our strategy by producing and characterizing heavily modified scaffold strands with amine groups for dye functionalization, phosphorothioate bonds for stability, and biotin for surface immobilization. We further validated our sequence design approach for precise conjugation of biomolecules by synthetizing scaffolds including binding loops and aptamer sequences that can be used for direct hybridization of nucleic acid tagged biomolecules or binding of protein targets.

## 1 Introduction

The DNA origami technique offers unprecedented precision for the design and assembly of discrete, biocompatible, and functional nanoarchitectures, ranging from 10 to a few hundred nanometers (nm) in size.^1−5^ The unique addressability of DNA origami nanoparticles (DNA-NPs) also enables the organization of biomolecules (e.g., proteins,^6−8^ peptides,^9^ and nucleic acids^10,11^), fluorophores,^12,13^ and metallic nanoparticles^14,15^ with nanoscale precision and controlled stoichiometry.^16^ This unique capability endows the DNA-NPs with unique properties and functions when compared to other nanoparticle materials, which has led to the development of several promising nanocarriers that could replace some of the more classical materials, such as liposomes and polymeric nanoparticles, that are traditionally used in various biomedical applications.^17,18^ For instance, DNA origami is used for label-free RNA detection,^19^ triggered cargo release,^20,21^ vaccine development,^22,23^ immune cell stimulation,^24,25^ cancer immunotherapy,^26,27^ enzyme cascade reconstitution,^28^ and analysis of dynamic molecular events.^29,30^

The assembly of DNA-NPs is usually accomplished via slow annealing of a long single-stranded DNA (ssDNA) scaffold with several complementary short ssDNA oligonucleotides called “staple strands”. The staple strands can carry specific functional groups, such as carboxy,^31^ amine,^32^ thiol,^33^ and biotin,^8^ among others. These functional moieties can be located either internally or at the 3′- and 5′-ends of the staple strands, which allow further precision in localization of the functional moieties onto the final folded structures. Although this method of functionalization is extremely simple and robust, staple strands only provide access to fewer than half of the permissible sites on DNA-NPs and are subject to the inherent limitations of the chemical synthesis process. The need for excess staple oligonucleotides in origami folding and the cost associated with using multiple modified oligonucleotides can be an issue for scaling up the production of functional DNA-NPs. Moreover, multiple purification steps are usually required to remove the excess nonreacted oligonucleotides prior to further modification, which might reduce the overall production yield. Therefore, when precisely located modification is not required and when the degree of functionalization is the only critical parameter (e.g., fluorophores for tracking and phosphorothioate backbone modifications for improving stability), using individually modified staple strands might not be the optimal solution. Thus, methods that can turn the scaffold strand into programmable component of DNA-NPs would significantly facilitate some applications of the DNA-NPs by accelerating the synthesis and reducing the overall costs associated to their production.

Using the ssDNA scaffold as a means for functionalizing DNA-NPs could simplify the assembly process and reduce the overall synthesis costs. However, this approach requires modified scaffolds to be synthetized and therefore does not allow the use of the commercially available DNA templates like the M13mp18 ssDNA circular plasmid commonly used for DNA origami folding. In recent years, only a few studies have demonstrated the production of functional DNA origami scaffolds, but these approaches are mainly focused on tuning the size and sequence of scaffolds to overcome the length limitation inherent to commercially available versions of M13mp18.^34−36^ Recently, a study by Chen et al. used bacteriophage genome modifications to introduce multiple aptamers at specific locations on folded structures.^37^ Although this method can generate a large amount of scaffold (greater than milligram quantity) and is highly efficient for the introduction of aptamers via sequence modification, bacteriophage production of ssDNA does not allow chemical functionalization and still requires the use of modified oligonucleotides to further functionalize the folded DNA-NPs.^37−39^

As an alternative, polymerase chain reaction (PCR)-based strategies, particularly the asymmetric PCR (aPCR) method, offer a higher flexibility in sequence design and sequence length and can be used for direct incorporation of functional groups into the scaffold during synthesis.^40−42^ The mechanism of ssDNA production via aPCR is based on using an asymmetric concentration of primers (e.g., 50× molar excess of the forward primer relative to the reverse primer),^43^ which biases replication toward one strand of the double-stranded DNA (dsDNA) template. This method allows for the direct synthesis as well as purification using a simple gel extraction technique of ssDNA from various templates, in comparison with other classic PCR-based strategies. Indeed, PCR methods generate dsDNA products that require extra steps to separate the two DNA strands prior to their use as a scaffold by employing strategies such as biotin-streptavidin capture and separation, strand-specific digestion, or polymer catch-and-release (SNAPCAR).^39^ The aPCR strategy has already been used to produce large quantities of kilobase-length ssDNA with custom sequences and incorporate chemical modifications via the introduction of substituted deoxynucleoside triphosphates (dNTPs).^42^ This method requires minimal optimization for the production of newly designed ssDNA scaffolds and can easily be scaled up^44^ without significant additional cost to support the production of multifunctional DNA-NPs by directly synthesizing modified scaffolds. However, to successfully implement a system for scaling up production, alternative purification methods should be utilized, such as different chromatographic techniques.^45^

**Figure 1:**
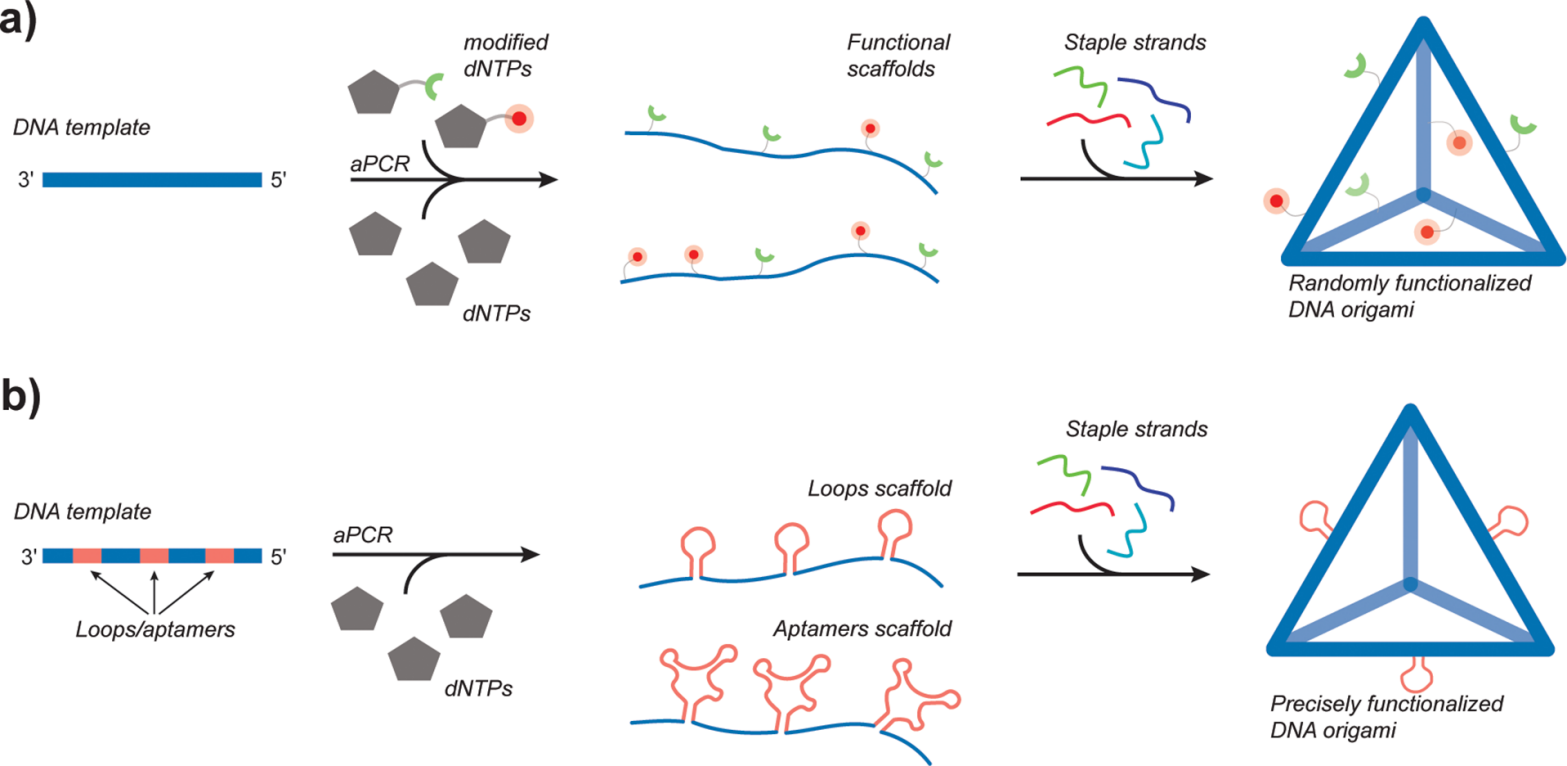
Schematic of the aPCR method used to produce site nonspecifically and specifically functionalized DNA-NPs. (a) Randomly functionalized scaffolds. The functionalization of DNA-NPs is achieved through nonspecific insertion of modified dNTPs during the aPCR. The modified scaffolds can be folded with regular staple strands to yield functionalized DNA-NPs. (b) Precisely functionalized scaffolds. Functionalization of DNA-NPs with sequence-specific design of scaffolds to site-specifically display binding loops and aptamers at precise locations on the folded structures.

The effect of simultaneously introducing multiple types of modified dNTPs on the yield of the aPCR has not yet, to our knowledge, been investigated. In addition, it is not clear to what extent the folding of DNA-NPs will be affected by heavily modified scaffolds. Here, we demonstrate the capability of aPCR to incorporate various ratios of normal and modified dNTPs within the scaffold to synthetize functionalized DNA-NPs that has biotin groups, amine groups, and/or phosphorothioate backbone linkages. These heavily modified scaffolds can be further folded into functional DNA-NPs (Figure 1, top panel) without the need for using functional staple strands. These functional scaffolds can also be chemically modified at a later stage, as we demonstrated using *N*-hydroxysuccinimide (NHS) ester conjugation for amine coupling. In addition, to allow for the precise attachment of biomolecules to functionalized DNA-NPs without using modified oligonucleotides, we also designed scaffolds with small anchoring stem loops and aptamers, as previously done with bacteriophage-based ssDNA production.^37^ These sequences, which are not participating in the folding of DNA-NPs, are displayed precisely on the folded nanoparticles by strategically inserting them into the sequence of the scaffold (Figure 1, bottom panel). They can be tested via binding of nucleic acid tagged biomolecules (ssDNA loop) or direct binding of biomolecules and cell targeting (aptamers). Furthermore, with this strategy, the user can perform additional functionalization of the DNA-NPs without purification steps for the prior removal of excess staple strands. The excess staple strands that can interfere with further applications are removed at the same time as the biomolecules that are conjugated to the DNA-NPs, which reduce the number of required purification steps, lead to a potential increase in the production yield, and lessen the overall cost of manufacturing.

## 2 Experimental Section

### 2.1 Materials

All DNA oligonucleotides (“staple strands”), aPCR primers, and gBlocks were purchased from Integrated DNA Technologies (IDT) in lyophilized form, resuspended in DNAse/RNAse free water, and incubated at 50 °C for 20 min to ensure proper resuspension. The concentration was measured using a NanoDrop One. The aPCR primers and staple strands were stored at −20 °C at a concentration of 500 μM, and the gBlocks were stored at −20 °C at a concentration of 10 ng/μL. All strands were directly used without further purification. All sequences used are listed in Tables S1–S10. Basic and low melting point (CAS no. 9012-36-6) agarose was purchased from IBI Scientific. Amicon Ultra-0.5 Centrifugal Filters (10 and 100 kDA MWCO) (cat. nos. UFC5010 and UFC5100) were purchased from Sigma Aldrich. The surface plasmon resonance (SPR) gold sensor chips (cat. no. SEN-AU-100-10) and all SPR reagents were purchased from Nicoya. The reagents 11-mercaptoundecanoic acid (CAS no. 71310-21-9), *N*-(3-dimethylaminopropyl)-*N*′-ethylcarbodiimide hydrochloride (EDC), *N*-hydroxysuccinimide (NHS), thrombin (CAS no. 9002-04-4), and biotinylated bovine serum albumin (BSA-Biotin) were acquired from Sigma-Aldrich. The Zymoclean Gel DNA recovery kit was purchased from Zymo Research (cat. no. D4008). The M13mp18 ssDNA template (cat. no. N04040S), the Lambda phage template (cat. no. N3011S), the 1 kb plus DNA ladder (cat. no. N0550S), the ultra-low range DNA ladder (cat. no. N0558S), and the OneTaq (OT) Hot Start DNA Polymerase (cat. no. M0481) were procured from New England BioLabs (NEB). The AccuStart Taq DNA Polymerase HiFi (HF) (cat. no. 95085-05 K) and AccuStart Long Range (AL) SuperMix (cat. no 95199-100) were purchased from Quantabio. The streptavidin was obtained from Genscript (cat. no. Z02043). Recombinant Cys-protein G was purchased from Prospec (cat. no. pro-1238). PNA-maleimide was provided by PNA Bio. All modified dNTPs: biotin-16-aminoallyl-2′-deoxycytidine-5′-triphosphate (cat. no. N-5002), 2′-deoxynucleoside alpha-thiol nucleotides (cat. nos. N-8001, N-8002, N-8003, and N-8004), and 5-aminoallyl-2′-deoxycytidine-5′-triphosphate (cat. no. N-2048), along with unmodified nucleotides (cat. nos. N-2510, N-2511, N-2512, and N-2513), were obtained from TriLink BioTechnologies. The Cy5-NHS linker was purchased from Nanocs (cat. no. S5-1, 2). The mammalian cell lysis kit for the stability assay was obtained from Sigma-Aldrich (cat. no. MCL1-1KT). HEK293T cells were acquired from ATCC.

### 2.2 DNA-NP Design

The DNA-NPs used in this study were designed with CaDNAno2^46^ (6-helix bundle [6-HB]), Tiamat,^47^ and DAEDALUS^3^ (pentagonal bipyramid [PB] and tetrahedron [Tet]) design programs. The 3D models of the 6-HB, the PB, and the two tetrahedra (31 and 42 nucleotide [nts] edge length) DNA-NPs were created using Chimera^48^ with the atomic coordinate file (.pdb) acquired from CanDo (6-HB), TacoxDNA,^49^ and DAEDALUS (tetrahedra and PB) (Figure S1). The scaffold and staple strand sequences are available in Tables S1–S10.

### 2.3 Scaffold Synthesis Using aPCR

#### 2.3.1 Scaffold Production with Nonmodified dNTPs

The ssDNA scaffolds were synthesized using the previously published aPCR protocol.^42^ The reaction mixture for the HF enzyme for a 50 μL reaction was as follows: 1 μM of forward primer, 20 nM of reverse primer, 0.5 μg/mL of M13mp18 ssDNA template (or 0.2 μg/mL for all the gBlocks or 0.1 μg/mL for the Lambda phage template), 200 μM of deoxynucleoside triphosphates (dNTPs), 1× HiFi PCR buffer, and 2 mM magnesium sulfate (MgSO_4_) with 1.25 U of the HF enzyme.

Alternatively, two other enzymes were also tested, namely, the OT and AL enzymes. The OT enzyme was used with the buffer provided by the vendor (5× OneTaq Standard Reaction Buffer), and the AccuStart Long Range SuperMix includes enzymes, buffer, and dNTPs altogether, so no additional dNTPs were added for the reactions with the latter enzyme. For the HF and OT enzymes, the aPCR was carried out in a Bio-Rad T100 Thermal Cycler using the following program: 94 °C for 1 min for the initial denaturation followed by 35 cycles of 94 °C for 20 s, 55 °C for 30 s, and 68 °C for 1 min per kilobase to be amplified.^3^ For the AL enzyme, the aPCR was carried out in the same Thermal Cycler with the following program: 95 °C for 3 min for the initial activation followed by 35 cycles of 92 °C for 30 s, 55 °C for 30 s, and 68 °C for 1 min per kilobase to be amplified.

#### 2.3.2 Scaffold Production with Modified dNTPs

For the direct synthesis of modified scaffolds, modified dNTPs were mixed at various concentrations with nonmodified dNTPs to a total dNTP concentration of 200 μM. The PCR programs used were the same as in the paragraph above. All modified scaffolds were produced using the HF enzyme.

### 2.4 ssDNA Scaffold Validation and Purification

The correct synthesis of the ssDNA scaffolds was validated with gel electrophoresis, and the ssDNA was further purified via gel extraction. Specifically, the products of the aPCR were loaded into a 2% low-melt agarose gel for the 449 nucleotide (nts) scaffold and all gBlocks, and 1% low melt agarose gel for the 1616 and 1644 nts scaffold, along with 1× Tris-acetate ethylenediaminetetraacetic acid (EDTA) buffer (TAE-buffer pH 8.0) prestained with 0.5 μg/mL ethidium bromide. A BioRad electrophoresis unit was used for agarose gel electrophoresis at 100 V at room temperature. Gel images were taken with an Azure c150 gel imaging workstation and analyzed with the ImageJ software^50^ to validate the efficiency of the reaction. The ssDNA recovery was performed with a Zymoclean Gel DNA recovery kit as previously described.^42^ The concentration of ssDNA was measured with a Nanodrop One (ThermoFisher). The ssDNA scaffolds were stored at −20 °C until further use.

### 2.5 Postsynthesis Scaffold Functionalization

Amino (NH_2_) modified ssDNA scaffolds were further modified using *N*-hydroxysuccinimide (NHS) ester coupling. Cyanine 5 (Cy5) fluorophore-NHS ester was conjugated to NH_2_-ssDNA scaffolds to obtain Cy5-ssDNA. In a 1 mL sample tube, 20 to 30 pmol of NH_2_-ssDNA was mixed with Cy5-NHS ester (8 mM) in dimethylformamide (DMF) at a 50-fold excess molar ratio to the estimated amino groups available on the scaffold for the reaction (considering 100% incorporation rate of modified dNTPs in the scaffold) and about 30% (v/v) DMF and 20% (v/v) of reaction buffer (100 mM HEPES pH 8.2). The volume of reaction was adjusted with Ultrapure DNA/RNA free water to a total of 100 μL, and the sample was left in the dark on a rocker at low speed overnight.

Following the overnight reaction, the sample was mixed with sodium acetate solution (3 M, pH 5.2) and absolute ethanol (−20 °C) to final concentrations of 8 and 70% (v/v), respectively, at a total volume of 300 μL. After 2 h at −20 °C, the solution was centrifuged at maximum speed and at 0 °C in a Sorvall ST 8 refrigerated benchtop centrifuge for 4 h followed by removal of the supernatant, two washes of the pellet with absolute ethanol at −20 °C, and centrifugation for 15 min after each wash. The supernatant was carefully discarded to prevent disrupting the pellet. The pellet was left to dry overnight, and the dried pellet was resuspended in water and stored at −20 °C prior further use.

### 2.6 DNA-NP Assembly and Purification

The DNA origami NPs were folded in a one-pot reaction according to the protocol previously published by Veneziano et al.^3^ Briefly, the scaffold was mixed with a 10× molar ratio of excess staple strands in a 1× TAE buffer that had been complemented with 12 mM MgCl_2_ and annealed overnight in a thermocycler programmed with a set temperature gradient as follows: 95 °C for 5 min, 80–75 °C at 1 °C per 5 min, 75–30 °C at 1 °C per 15 min, and 30–25 °C at 1 °C per 10 min. After folding, the DNA-NPs were purified and concentrated using 100 kDa Amicon Ultra centrifugal filters. The concentration of the purified DNA-NPs was measured by NanoDrop. Folded DNA-NPs were analyzed in a 1–1.5% agarose gel prestained with ethidium bromide ran at 100 V for 30 min and imaged with the Azure c150 imager.

### 2.7 Functionalization of DNA-NPs Folded with Scaffolds Produced from gBlocks

The procedure described in Section 2.6 was used to fold DNA-NPs with scaffolds produced with the different gBlocks. For functionalization with protein G (PG), PG was first conjugated with PNA-maleimide (Mal-GGK-cagtccagt-K) via the free cysteine located at its N-terminal, as previously described by Oktay et al.,^23^ at a 1:3 molar ratio of PG to PNA-maleimide. The PG-PNA product was purified from excess PNA via Amicon filter columns (10 kDA MWCO). The gBlock-derived DNA-NPs with loops carrying either complementary sequence for PNA hybridization or thrombin binding sequences were incubated with proteins (twofold excess PG-PNA or one- to two- or threefold excess of thrombin) at 37 °C for 1.5 h.

### 2.8 Atomic Force Microscopy (AFM) Characterization of the DNA-NPs

AFM characterization of assembled DNA-NPs was performed in fluid tap mode on a JPK Instruments NanoWizard 4 fast-scan AFM using USC-F0.3-k0.3 cantilever tips (NanoWorld). DNA-NPs were diluted to approximately 4 nM in 0.2 μm-filtered 0.5× Tris-borate EDTA (TBE buffer: 50 mM Tris base pH 8.3, 50 mM boric acid, 1 mM EDTA) with 12.5 mM MgCl_2_. The diluted NP sample (15 μL) was deposited onto a freshly cleaved disk of mica mounted on a metal puck and incubated for 5 min. Following that, 100 μL of the filtered buffer was added to the mica and then wicked off the surface with a lint-free optic wipe to remove any free DNA. This rinsing step was repeated once more, and then the sample was transferred to the AFM. For imaging, 100 μL of the filtered buffer supplemented with 5 mM NiCl_2_ was added to the mica. Images of 1 × 1 and 2 × 2 μm^2^ were acquired with 1000 pts/line and 1000 lines/scan at a rate of 8 and 4 Hz, respectively. Topography images were leveled and corrected for scanning artifacts in postprocessing using the open-source Gwyddion^51^ SPM software.

### 2.9 Fluorescence Measurements

#### 2.9.1 Quantification of NH_2_ Incorporation via Cy5-NHS Ester Reaction

A Tecan Safire^2^ Microplate Reader was used to measure the fluorescence intensity of Cy5-labeled ssDNA scaffolds with a fixed excitation at a wavelength of 590 nm, and the emission was measured from 640 to 700 nm. We used free Cy5-NHS solutions with concentrations ranging from 0 to 15 μM to prepare a standard curve for accurate quantification of Cy5 in our samples.

#### 2.9.2 Stability Assessment in 20% Mouse Serum via Time-Resolved Förster Resonance Energy Transfer (FRET)

The unmodified PB DNA-NPs and PB DNA-NPs containing scaffolds that were produced using 10, 20, and 35% phosphorothioate-modified linkage (henceforth referred to as “αThiol”) as well as the multifunctional PB (multi-V: 7.5% biotin, 15% NH2, 15% αThiol) were assembled in the presence of either only FAM (5′-donor)-modified staple strands or staple strands having both FAM and TAMRA (5′-acceptor) together (Table S4). FAM and TAMRA staple strands have been positioned to ensure a distance of approximately 3 nm between the donor and acceptor to maximize FRET efficiency as previously used.^23^ Following purification using 100 kDa Amicon columns as described in Section 2.6, fluorescently labeled DNA-NPs were incubated in PBS that had been complemented with 20% mouse serum. The degradation rates of the different DNA-NPs were tracked in a time-dependent FRET assay using a Tecan Safire^2^ Microplate Reader at a fixed wavelength excitation of 455 nm and emission reading from 500 to 700 nm. At the 10 h time point, DNase was added to achieve complete degradation of all DNA-NPs to be used as a reference. The stabilities of the DNA-NPs were calculated according to the changes in fluorescence intensity of the donor dye. The relative intensities of the donor dye from donor only FAM-containing PB (*I*_D_) and from FAM-donor/TAMRA-acceptor pair-containing PB (*I*_DA_) were subtracted from each other to determine the FRET efficiency (*E*). The formulas below were taken from Wei et al. (2013):^52^
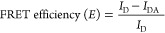


In accordance with the FRET efficiency, the fractions of folded DNA-NPs (denoted by θ) were then calculated using the following formula after treating NPs with DNase to complete degradation. Emax defines the initial (maximum) intensity difference at 0 min in serum between NPs with only FAM and with FAM/TAMRA together. Emin indicates the minimum intensity difference upon degradation in serum and DNase.
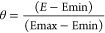


#### 2.9.3 Stability Assessment in Cell Lysate via Time-Resolved FRET Study

PBs assembled using unmodified and 20% αThiol-modified scaffold strands were prepared to contain the donor dye only (FAM) or donor and acceptor dyes (FAM-TAMRA). A cell lysis solution was prepared containing 50 mM Tris–HCl, 150 mM NaCl, 0.1% SDS, 0.5% deoxycholic acid, 1.0% Igepal CA-630, and protease inhibitor cocktail at a ratio of 1:100. HEK293T cells were grown in a T25 cell culture flask to achieve an approximate cell count of 10^6^. Cells were then washed twice with Dulbecco’s phosphate-buffered saline (DPBS) and treated with 1 mL cell lysis buffer. Cells were incubated at 4 °C on an orbital shaker for 15 min thereafter. The lysed cells were then scraped and collected in a 1.5 mL microcentrifuge tube, and the resulting lysate was centrifuged at 4 °C for 10 min at 12,000*g*. The supernatant was collected and stored at −20 °C until use.

Following purification, fluorescently labeled NPs were treated with the cell lysate from HEK293T cells, and a 12 mM MgCl_2_ 1× TAE buffer was used as a control. The degradation rates of the different NPs at 12 nM were tracked in a time-dependent FRET assay using a Tecan SparkControl Magellan Microplate Reader at a fixed excitation wavelength of 455 nm and emission reading at 524 nm (FAM emission maxima) and 594 nm (TAMRA emission maxima) for 24 h at 37 °C with recordings taken every 10 min. After subtracting background fluorescence (fluorescence from the sample containing the cell lysate only), the stability of NPs was calculated according to the changes in the fluorescence intensity of the donor dye. The relative intensities of the donor dye from only FAM-containing PB (*I*_D_) and from FAM/TAMRA pair-containing PB (*I*_DA_) were used to determine FRET efficiency (*E*) using the formula stated in the previous section.

### 2.10 Surface Plasmon Resonance Binding Assay

Surface plasmon resonance (SPR) binding experiments were carried out in a Nicoya Open SPR device. The bare gold sensor chips were first modified with 11-mercaptoundecanoic acid (2.2 mg/mL) in absolute ethanol for 48 h at room temperature to form a self-assembled monolayer (SAM) of carboxylic acid. Biotinylation of the SAM layer was achieved via amine-coupling reaction. Briefly, a mix of 50 μL of EDC (200 mM) and 50 μL of NHS (50 mM) was added to the sensor surface for 3 min. Afterward, the surface was tilted to remove the EDC/NHS solution, and then 100 μL of biotinylated bovine serum albumin (BSA) (0.5 mg/mL) was immediately added on top of the sensors for 3 min of incubation. In the SPR device, 1 mg/mL streptavidin was immobilized onto the biotinylated surface using a flow rate of 20 μL/min, and then 30 nM of biotinylated DNA-NPs was injected and flowed over the surface at a flow rate of 20 μL/min. A solution of 10 mM glycine-HCl pH 2.4 was used for regeneration at a flow rate set to 150 μL/min. For all SPR experiments, we use the following running buffer: phosphate-buffered saline (PBS pH 7.4, 137 mM NaCl, 2.7 mM KCl, 8 mM Na_2_HPO_4_, and 2 mM KH_2_PO_4_) supplemented with 0.005% (v/v) Tween 20. Tween 20 was added to the running buffer to avoid nonspecific adsorption of the biomolecules to the tubing and the flow cell.

### 2.11 Endotoxin Assay

Endotoxin assays were performed using the ToxinSensor Chromogenic LAL Endotoxin Assay Kit from GenScript following the vendor instructions and with a measurable concentration range between 0.01 and 1 endotoxin unit (EU)/mL. This kit is designed for *in vitro* quantification of endotoxin presence via chromogenic detection with a modified limulus amebocyte lysate (LAL) and synthetic color-producing substrate. According to the protocol, well-mixed test samples were diluted up to 100 μL with LAL Reagent Water in sterile cuvettes, and an equal volume of reconstituted LAL was added to each vial. Following a pretimed 37 °C incubation on heating blocks, 100 μL of chromogenic substrate solution was added prior to an additional heated incubation for 6 min. Finally, 500 μL of a stop solution and two different color stabilizers were sequentially mixed into the samples before their absorbance was read at a wavelength of 545 nm in a 96-well plate. These values were then compared to a standard curve that had been generated from endotoxin standards within the desired concentration range to graphically determine the endotoxin content of the test samples.

### 2.12 Statistical Analysis

Data are shown as mean ± standard deviation on all the graphs. All experiments were performed at least in triplicates. Mean comparisons of more than two groups were calculated using one-way or two-way analysis of variance (ANOVA) depending on the number of variables. Following ANOVA, a Tukey post hoc test was applied as a multiple comparison test. All statistical analyses were performed in Microsoft Excel, RStudio, and GraphPad Prism.

## 3 Results and Discussion

### 3.1 Scaffold Production by aPCR Using Different Taq Polymerases

**Figure 2:**
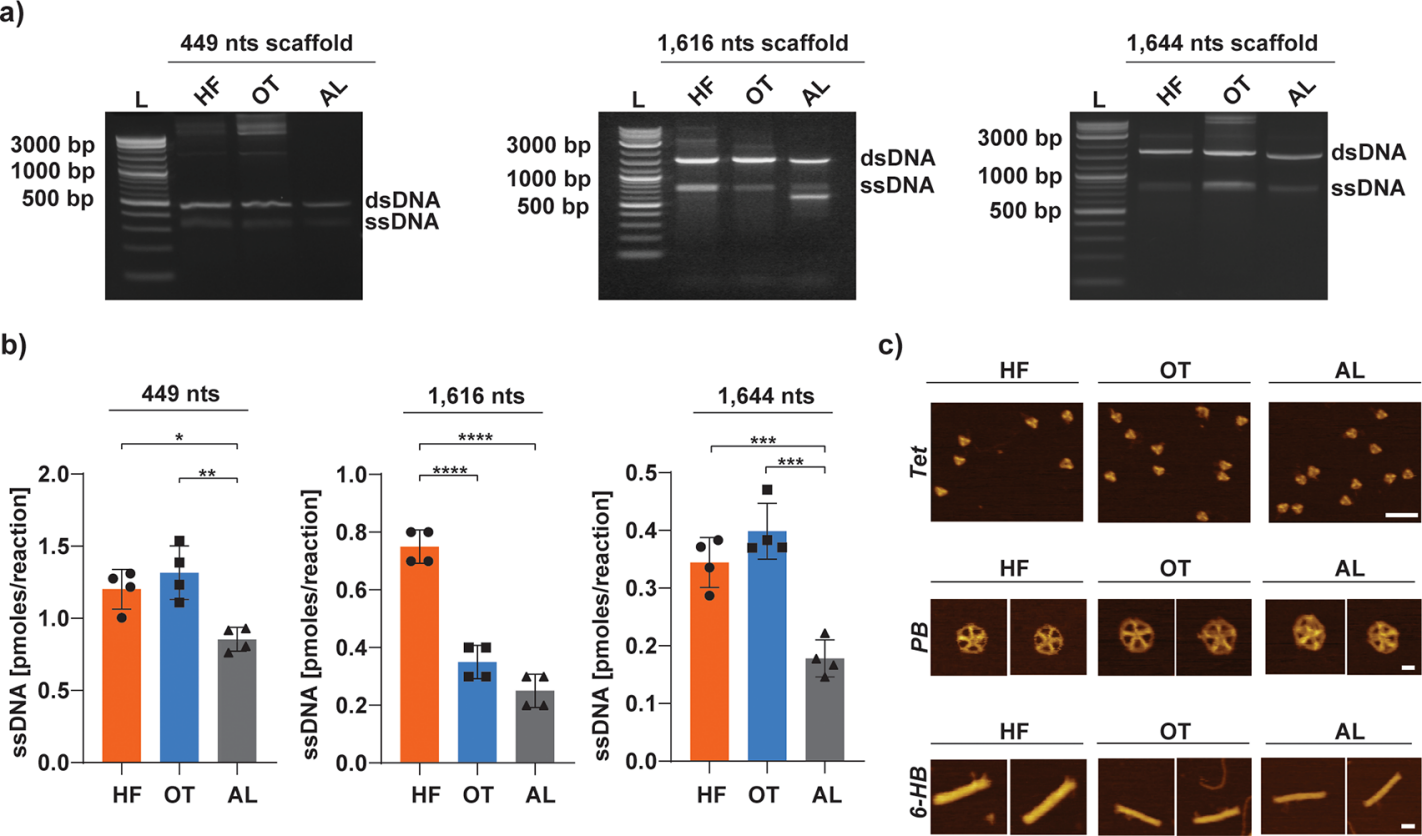
DNA origami scaffold production with three different Taq polymerases. (a) Representative gels for aPCR production of unpurified ssDNA scaffolds in lengths of 449 nts (*left*), 1616 nts (*middle*), and 1644 nts scaffold (*right*) using different Taq enzymes (HF, OT, and AL). (b) ssDNA production yield calculated for the three different enzymes tested. Error bars represent standard deviation of the mean (*n* = 4 independent samples/group). The *p* values are from a one-way ANOVA and a Tukey post hoc test (*p < .05, **p < .01, ***p < .001, ****p < .0001). (c) AFM topography images of the different DNA-NPs folded with the scaffolds produced by the three enzymes (scale bar: 50 nm for Tet; 20 nm for PB and 6-HB).

Polymerases with either a reduced or lack of 3′ to 5′ exonuclease activity, like *Taq* polymerases, are well-suited to produce ssDNA of up to 15 kb in length via aPCR.^42^ Because aPCR scaffold synthesis is highly dependent on the availability of specific enzymes, it is important to avoid relying on only one commercially available polymerase source. Therefore, in addition to the HF enzyme that was previously used,^3,42^ we evaluated two other commercially available enzymes with similar exonuclease activities to determine their applicability in pure ssDNA scaffold synthesis, namely, the OT and the AL enzymes. To assess their capacity to produce ssDNA and compare their production yield with HF, we amplified the three different ssDNA scaffolds (449, 1616, and 1644 nts in length) using the general aPCR protocol that had previously been optimized for the HF enzyme.^42^ The results presented in Figure 2a demonstrate that all three enzymes tested are able to produce ssDNA with minimal byproducts for all three sequence lengths amplified. After gel purification, we evaluated the production yield for all three enzymes. Figure 2b shows that OT and HF enzymes yield a similar quantity of the 1644 nts ssDNA scaffold with an average 0.39 and 0.34 pmol per 50 μL reaction, respectively. Although this result was informative about our enzyme efficiency, the reaction was not yet optimized as it was for the HF enzyme in our previous study.^42^

We thus explored if the OT enzyme efficiency could be improved by changing key parameters of the reaction conditions as was previously done with the HF enzyme.^42^ We first replaced the original OT standard buffer with the HF buffer that had been complemented with various concentration of MgSO_4_ ranging from 1 to 4 mM*.* Using these conditions, we were able to succesfully produce the 1616 and 1644 nts ssDNA scaffolds with the OT enzyme as seen on Figure S2a. We estimated the ssDNA production yield by analyzing the band brightness from gel electrophoresis with ImageJ (Figure S2b) and determined that 1.5 and 2 mM MgSO_4_ produced the highest quantity of ssDNA for both 1616 and 1644 nts, which is similar to the optimal conditions determined for the HF enzyme.^42^ The difference between these two MgSO_4_ concentrations was not significant, with 1.01 pmol per 50 μL reaction for the 1616 nts scaffold at 1.5 mM MgSO_4_ and 0.98 pmol per 50 μL reaction with 2 mM MgSO_4_. Similarly, the 1644 nts scaffold resulted in 0.66 pmol per 50 μL reaction with 1.5 mM MgSO_4_ and 0.79 pmol per 50 μL reaction with 2 mM MgSO_4_. Next, we compared this optimized OT reaction condition of 2 mM MgSO_4_ on the production yield of the original OT enzyme and buffer. Figure S2c shows that a higher yield of production is achieved with the HF buffer with 0.96 pmol per 50 μL reaction for the 1616 nts scaffold vs 0.35 pmol per 50 μL reaction in the OT standard buffer. The same improvement was observed with the 1644 nts scaffold with a quantity of 0.80 pmol per 50 μL reaction for the HF buffer vs 0.2 pmol per 50 μL reaction using the OT standard buffer. Interestingly, the results obtained for OT with the HF buffer complemented with 2 mM of MgSO_4_ are ∼3 and ∼4 times higher than the quantity produced with the HF enzyme for 1616 and 1644 nts scaffolds, respectively (Figure 2 and Figure S2c).

We further assessed the importance of the final extension step in the aPCR with the OT enzyme using the optimized conditions we determined earlier and for the 1616 nts scaffold. Indeed, final extension is generally used in classic PCR to allow final extension of the amplicons and allow reannealing of the complimentary strands of DNA. However, because aPCR is biased toward ssDNA production, this step might not be necessary and could result in degradation of the ssDNA products. Using ImageJ, we analyzed the quantity of ssDNA products after the PCRs (Figure S2d) and determined that the amount produced was significantly higher when not using the final extension, which could be caused by the degradation of ssDNA occuring in the final extension step due to the proofreading activity of the polymerase.

We then evaluated the potential use of the AL enzyme (adapted for amplification of long fragments). Our results show that we obtained a higher efficiency of OT and HF for the amplification of a short 449 nts fragment with an average of 1.32 and 1.2 pmol per 50 μL reaction, respectively, both higher than the 0.85 pmol produced with the AL enzyme. Likewise, the AL enzyme generated a lower amount of 1644 nts scaffold in comparison with the two other enzymes at only 0.18 pmol per 50 μL reaction. Similarly, the yield of ssDNA scaffold produced with the AL enzyme was the lowest compared to the yield of scaffold produced with HF and OT enzymes, which was 0.7 pmol for HF, 0.4 pmol for OT, and 0.2 pmol for AL for the 50 μL reaction. This reduced yield might be due to its optimization for longer scaffolds as observed with the longAmp enzyme previously tested^42^ and shown in Figure S3a for amplification of 10 and 15 kb scaffolds. Moreover, as previously shown for the HF enzyme,^42^ the OT enzyme is not able to produce 10 and 15 kb scaffolds (Figure S3b), which was expected given the limitations in amplification for this enzyme.

PCRs are highly sensitive to various factors such as the sequence, the length, and the enzyme used; thus, our results showing variation between the sequences produced are not unexpected. The decrease in yield can be largely attributed to the accumulation of partial products from repeated cycles of amplification that are unable to serve as substrates for additional cycles. Greater amounts of ssDNA are typically produced for our tested short (449 nts) and mid-length (1616 and 1644 nts) scaffolds as a result of the overall efficiency of PCR methods, which can be impacted by critical design factors like the choice of primers and sequence composition. More specifically, as previously reported,^40^ special consideration should be taken to reduce the exponential amplification of off-target dsDNA sequences, limit high GC-content, and avoid long repeated regions or long complementary regions. In comparison, other PCR-based methods for ssDNA production that rely on strand separation (e.g., streptavidin-coated magnetic beads) have limitations in terms of yield of ssDNA due to the extra steps of separation of ssDNA from dsDNA.^53−55^ In sum, these data support that the OT enzyme is a good alternative to the HF polymerase. The AL enzyme, although less effective for small fragments, may be considered better-suited for longer scaffolds.

### 3.2 Folding of ssDNA Scaffolds into DNA-NPs

To ensure that the scaffolds produced by these enzymes could be properly folded into DNA-NPs, we assembled the 449 nts (31 and 42 nts edge length) scaffold into Tet, 1616 nts (52 nts edge length) scaffold into PB, and 1644 nts scaffold into an 80 nm long six-helix bundle (6-HB).^25^ The gel electrophoresis presented in Figure S4 shows that the scaffolds produced by the three different enzymes are pure and can be folded in the presence of an excess of staple strands. The proper folding into the Tet, PB, and 6-HB rod was confirmed with AFM for all enzymes (Figure 2c and Figures S5–S8).

### 3.3 Nonspecific Functionalization of DNA-NPs

We used the M13mp18 circular single-stranded plasmid as our aPCR template for the production of ssDNA scaffolds of lengths 449, 1616, and 1644 nts. The modification sites that were targeted include the cytosine (C) nucleotides for the amino and biotin modifications and all nucleotides for the phosphorothioate modification. These modifications were directly introduced within the scaffolds by systematically substituting the normal nucleotide precusors (dCTPs for amine and biotin and dNTPs for phosphorothioate) with the corresponding functionalized analogues in the aPCR mix. The three amplified regions of the M13mp18 that formed the scaffolds of 449, 1616, and 1644 nts contained about 22, 20, and 24% C bases, respectively, thus offering a great number of possible sites for functionalizing the scaffolds and fine-tuning the final incorporation concentration of these chemical modifications.

#### 3.3.1 Single Type of Modification

Leveraging the capability of the aPCR method to allow incorporation of modified dNTPs to introduce nonspecific modifications in the scaffold can drastically reduce the cost of DNA-NP production while also enabling easy tuning of the number of modifications and keeping staple sites available for other functional groups. We tested the incorporation of three modified dNTPs (NH_2_-dCTPs, biotin-dCTPs, and αThiol-dNTPs) individually or in combination at different ratios. The effect of incorporating different ratios of modified dNTPs in the aPCR as well as using a combination of multiple modified dNTPs is presented in the subsections below. The following sections refer to the percentage modification with amino and biotin groups as the number of C bases relative to the total number of C bases in the entire scaffold that were substituted with modified dCTPs. For αThiol, the modification percentages indicate the percentage of substitution for all four dNTPs used to synthetize the scaffolds. As an example, 10% dCTP modification refers to only 10% of the C bases among all C bases of the scaffold as modified while all other dNTPs remain nonmodified. However, 10% αThiol modification signifies that 10% of all dNTPs (i.e., A, C, G, and T bases) were modified accordingly. All modifications were performed with the HF enzyme.

##### 3.3.1.1 Amino Modification

We first synthesized NH_2_-ssDNA scaffolds (1644 nts) with different percentages of NH_2_ groups (0, 10, 20, 50, 75, and 100%) by systematically substituting a fraction of dCTP with the NH_2_-dCTP analogue in the aPCR mix. For example, the 10% NH_2_-ssDNA scaffold was prepared using a 10:90 ratio of NH_2_-dCTP/dCTP monomer in the aPCR. The efficacy of the aPCR was determined by performing agarose gel electrophoresis for the different ratios tested (Figure S9a). From 0 to 75%, the aPCR efficiency appeared similar with a slight decrease in the ssDNA band intensity for 75% (Figure S9a). Interestingly, as previously shown for 100% substitution of canonical dNTPs with αThiol-dNTPs^42^ or biotin-dCTPs,^41^ replacing the dCTPs entirely with NH_2_-dCTPs (representing the 100% NH_2_-ssDNA scaffold sample) also completely inhibited the aPCR.^42^ As observed in the gel electrophoresis, the production yields calculated after purification confirmed that a decrease in the quantity of ssDNA produced correlated with an increase in the ratio of NH_2_-dCTP used (Figure S9b). The aPCR with the ratios of 0, 10, 20, and 50% modified dCTPs yielded ∼0.95, ∼0.97, ∼0.83, and ∼ 0.84 pmol ssDNA scaffolds per 50 μL of aPCR, respectively. The mean differences of the yield of ssDNA scaffolds were shown to not be statistically significant (Figure S9b). A statistically significant decrease was observed in the quantity of scaffold produced from the reaction using 75% NH_2_-dCTPs. Reactions with 75% NH_2_-dCTPs resulted in ∼25% decrease for the 1644 nts length scaffold compared to the 0% modified scaffold (0.71 pmol per reaction). The aPCR for the production of the 1616 nts length scaffold with varying percentages of NH_2_-dCTPs also showed that replacing all dCTPs with NH_2_-dCTPs results in a sharp decrease in the yield of the ssDNA product (Figure S9c). Quantitative data obtained from the purified ssDNA scaffold with five different percentages of NH_2_ modification were recorded as 0.33 pmol for 10%, 0.25 pmol for 20%, 0.45 pmol for 50%, and 0.42 pmol for 75% NH_2_ modified scaffold (Figure S9d). As for the 1644 nts scaffold, replacing 100% of the dCTPs by NH_2_-dCTPs led to ssDNA production that was not sufficient to be visualized on our gel and therefore not purifiable.

The same downtrend was observed with the short 449 nts ssDNA scaffold when increasing the ratio of modification (Figure S9e,f). The amount of ssDNA synthesized per reaction for each scaffold (0, 10, 20, and 50%) was calculated as 1.81, 1.85, 1.69, and 1.72 pmol, respectively. As for 1644 nts, there was no statistically significant difference among the yield of scaffolds with modifications from 0 to 50%. However, performing the reaction with 75 and 100% NH_2_-dCTPs lowered the yield by 36 and 98%. The total amounts of ssDNA produced after reaction with 75 and 100% were ∼1.15 and ∼0.04 pmol, respectively. In a different set of experiments, the OT enzyme was also used for the synthesis of NH_2_ modified scaffolds to confirm that the enzyme is also able to synthetize modified scaffolds as for the HF enzyme (Figure S10).

**Figure 3:**
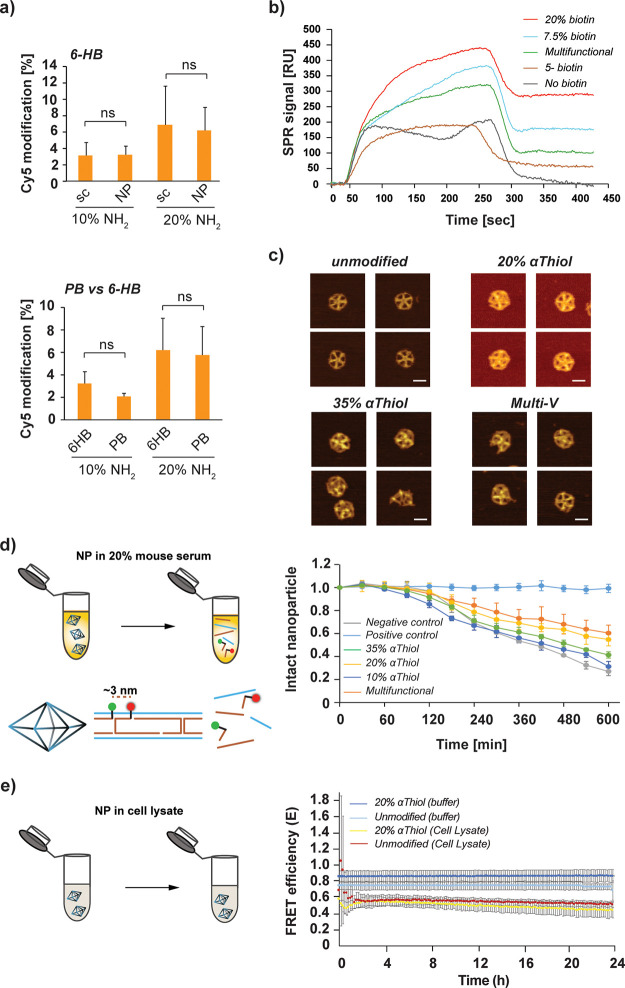
Characterization of purified DNA origami NPs folded with modified scaffolds. (a) NH_2_ quantification of modified scaffolds (1644 nts) and NPs (6-HB and PB) via labeling with Cy5-NHS (ns: nonsignificant data based on Student’s *t* test). (b) Representative SPR curves showing the binding profile of NPs with various numbers of biotin provided by scaffold or staple strands and the nonspecific binding profile of NP without modification used as a negative control. (c) AFM images of unmodified PB (upper left), 20% αThiol PB (upper right), 35% αThiol PB (bottom left), and multifunctional PB NP (denoted by “multi-V” that is formed by 7.5% biotin, 15% NH_2_, and 15% αThiol modification containing scaffold) (bottom right) (scale bar: 20 nm). (d) The FRET assay was used to assess the stability of NPs with different percentages of αThiol modifications. Data in the bar graphs display error bars that indicate the standard deviation of the mean (*n* = 3 independent samples/group). Statistical analyses were performed using two-way ANOVA and Tukey post hoc test. (e) The viability of NPs with 20% αThiol modifications vs unmodified scaffolds in the HEK293T cell lysate or TAE buffer only for *in vitro* and *in vivo* applications was also tested via FRET assay. Time-course graphs display error bars that indicate the standard deviation of the mean (*n* = 3 independent samples/group).

To validate the incorporation of amino group in the scaffold, we coupled 1644 nts NH_2_-ssDNA scaffolds produced with 10 and 20% NH_2_-dCTP with Cy5-NHS ester overnight. Using a modified procedure typically used for ssDNA oligonucleotide precipitation and purification as described in Section 2.5, we were able to purify and recover around 80% of the modified 1644 nts long ssDNA after conjugation with Cy5-NHS. After complete drying and resuspension of the Cy5-labeled ssDNA scaffolds, we estimated the number of Cy5 dyes incorporated per scaffold using fluorescence spectroscopy (Figure S11). Our results show that we were able to successfully incorporate Cy5 in a concentration-dependent manner and make fluorescently labeled scaffolds (Figure S11). In addition, we also performed the same Cy5-NHS labeling after assembling 6-HB NPs with the 10 and 20% NH_2_-modified scaffolds. The results in Figure 3a show that the Cy5 labeling efficiency for scaffold only and the formed-NPs was comparable but also NH_2_-dCTP concentration dependent; in both cases, ∼3%, or ∼12 Cs, were NH_2_-functionalized in the 10% NH_2_-dCTP samples, and ∼6%, or ∼24 Cs, were NH_2_-functionalized in the 20% NH_2_-dCTP samples. The same result is obtained with a different scaffold used to fold a pentagonal bipyramid (abbreviated as PB, 1616 nts scaffold with about 21% of C content) that shows a similar proportional incorporation of Cy5 when doubling the ratio of NH_2_-dCTP used (Figure 3a, right panel) with 2% (∼7 Cs) and 6% (∼19 Cs) for the reactions using 10 and 20% of NH_2_-dCTP, respectively.

##### 3.3.1.2 Biotin Modification

To produce NPs that can be used for surface immobilization, streptavidin coupling, and/or affinity-based purification, we produced biotinylated ssDNA scaffolds using various concentrations of biotin-dCTPs as previously published.^42^ 6-HB NPs were folded with either the biotinylated scaffolds (1644 nts long) or the nonbiotinylated scaffold and then coupled with streptavidin. As a positive control, we prepared 6-HB NPs with five biotinylated staple strands (Table S5) and the nonmodified scaffold. The gel image in Figure S12 shows that DNA-NPs could bind to streptavidin whether they have been assembled with the biotinylated staple strands or the biotinylated scaffold. For the biotinylated scaffolds, the binding was dependent on the percentage of biotin-dCTPs used, and thus, NPs folded with 20% biotinylated scaffold led to more streptavidin binding than 5 and 10% biotinylated scaffold and 5-biotinylated staple containing NPs. It is important to note that we use a large excess of streptavidin in this experiment based on the amino modifications and dye incorporation, which gives an estimate of available sites for further binding with streptavidin. Indeed, modifying ssDNA scaffolds with a large number of biotin functional groups (e.g., 20% biotinylation) requires using a large excess of streptavidin to avoid clustering of multiple particles. Therefore, users are encouraged to determine the number of biotin sites prior to functionalization or to use the same conservative approach as us with a larger excess of streptavidin (Figure S12).

Using SPR, we demonstrated the accessibility of the biotin groups by binding the PB NPs onto a streptavidin-immobilized gold surface (Figure 3b and Figure S13). PB NPs (folded with 7.5 and 20% biotinylated scaffold) were compared with the bare PB NP and the PB folded with biotinylated staple strands and unmodified scaffold. The maximum binding was observed for the PB folded with the 20% biotin scaffold. Interestingly, the PB folded with the 7.5% biotin scaffold exhibited a binding similar to the PB folded with five biotin-staple strands. This clearly demonstrates our capability to tune the quantity of biotin on the scaffold, which could be very useful for immobilization or capture of biotinylated molecules via streptavidin coupling. Also, these results confirmed that biotinylated sites on the scaffold were still accessible in assembled NPs.

##### 3.3.1.3 αThiol Modification

To assemble nuclease-resistant DNA-NPs, we synthesized ssDNA scaffolds (449, 1616, and 1644 nts) using phosphorothiate-modified dNTPs (αThiol-dNTPs) at different ratios (0, 10, 20, 50, and 75%). In this case, normal dNTPs were proportionately and equally altered in the aPCR mixtures to achieve the desired αThiol-dNTP ratios because the substitution of canonical dNTPs with phosphorothioate analogues was aimed to be random. Agarose gel electrophoresis (Figure S14a) was used to compare each modified 1644 nts scaffold. The reaction with the ratios of 0, 10, and 20% αThiol-dNTPs yielded ∼0.83, ∼0.71, and ∼0.77 pmol ssDNA scaffolds per 50 μL of aPCR, respectively (Figure S14b). We were not able to quantify the yield when using 100% αThiol-dNTPs because the production yield was not enough to detect any ssDNA before purification. This was supported by a previous study showing that replacing all phosphodiester bonds in the DNA backbone with phosphorothioate was not successful with aPCR.^42^ The yields for 50 and 75% modified scaffold decreased by ∼51 and ∼66% with respect to the 0%, and we obtained 0.4 and 0.29 pmol per 50 μL aPCR from 50 and 75% αThiol scaffolds, respectively (Figure S14b).

We further tested the efficiency of incorporation of phosphorothioate linkages for the 449 and 1616 nts scaffolds. The gel image of the 449 nts scaffold in Figure S14c shows a significant decrease in the band intensity of the ssDNA scaffolds when using ratios of αThiol-dNTPs greater than 50% in the reaction. The yields for the 449 nts ssDNA scaffolds with 0, 10, and 20% αThiol-dNTPs were 1.94, ∼1.87, and 1.79 pmol per 50 μL of aPCR, respectively (Figure S14d). We obtained 1.39 pmol 50% αThiol modified scaffold per reaction, which represents ∼29% decrease in the yield with respect to the unmodified scaffold. The relative yield decreased by ∼77 and ∼98% for 75 and 100% scaffolds in comparison to the unmodified scaffold, which corresponded to 0.46 and 0.04 pmol per 50 μL of aPCR, respectively. The gel for 1616 nts scaffold modification showed a decreasing pattern in each increasing percentage of modification (Figure S14e). The yield of the 1616 nts scaffolds modified with 10, 20, 50, and 75% αThiol was recorded as 0.46, 0.32, 0.29, and 0.10 pmol per 50 μL reaction, respectively (Figure S14f). Additionally, we included 35% αThiol modification in this set of experiments to evaluate the yield between two different percentages of modifications (20 and 50%). Based on that, the yield was calculated as 0.42 pmol per 50 μL reaction.

To assess the effect of using various percentages of αThiol modification on the folding and stability of the NPs, we performed a FRET assay on the PB NPs (see Section 2.9.2) based on a recent study.^23^ We folded PB NPs with 10, 20, and 35% αThiol modified scaffolds. Using AFM, we examined whether NPs were properly folded or not (Figure 3c and Figure S15). AFM images demonstrated that increasing the number of αThiol-dNTPs hampered the efficiency of folding. In particular, we observed that scaffolds synthetized with αThiol-dNTPs greater than 20% ratio (i.e., 35 and 50%) (Figure 3c and Figure S15) had less efficient folding, indicated in AFM images by the presence of unfolded scaffolds. On the basis of these results, we prepared and purified multiple PB samples with different percentages of αThiol-dNTPs (10, 20, and 35%) and assessed their stability with a FRET assay in 20% mouse serum (Figure 3d). Two staple strands pairs within the PB NPs were modified to incorporate two FAM dyes and two TAMRA dyes to use FRET signal decrease as a probe for monitoring structural destabilization in the NPs. The FRET signals from DNA-NPs (one prepared with FAM only and one with both FAM and TAMRA) were monitored upon incubation in 20% of mouse serum, and the results were compared with unmodified NPs in serum and in PBS for a 10 h period. After 10 h, the samples were treated with DNase to fully degrade the NPs and measure the minimum FRET signal expected. The change in FRET suggested that, although the stability of NPs folded with the 10% αThiol-dNTP scaffold was ∼4% higher than that of the unmodified NPs, incorporating 20% of αThiol modification increased the stability to about 28% over the course of the experiment with a percentage of intact particles of 55 vs 27% after 10 h. This means that the number of intact particles after 10 h almost doubled when using only 20% of αThiol-dNTPs. As expected from the agarose gel electrophoresis results and the AFM data, the 35% modification showed lower stability than 20% and resulted in 40% intact NPs after 10 h. The latter result also corroborates the previous observation we have made with particles folded with the scaffold containing 50% of αThiol-dNTPs that they do not fold properly and succumb more readily to serum degradation (Figure S15d).

#### 3.3.2 Viability of αThiol-Modified DNA-NPs *In Vitro* and *In Vivo*

For downstream applications requiring introduction of DNA-NPs into mammalian cells, we tested the stability of PB NPs in the cell lysate when assembled using the 20% αThiol scaffold vs the unmodified scaffold strand. As described in Section 2.9.3, the cell lysate from human embryonic kidney (HEK293T) cells was prepared, and dye-labeled PB NPs were incubated for 24 h in a time-resolved FRET assay. Results, summarized in Figure 3e, show that PB NPs remained stable regardless of the scaffold used for their assembly. Previous studies on the cytosolic stability of polyhedral DNA-NPs^56^ as well as in the cell lysate agree with these results.^57^

#### 3.3.3 Incorporation of Multiple Modifications

The aPCR protocol also offers the possibility to incorporate multiple modified dNTPs in various combinations and thus affords multiple functionalities in one DNA-NP for tracking, binding, or increased stability. After successfully demonstrating the incorporation of individual functional groups in the scaffold synthesis, we tested the incorporation of two or three distinct functional groups into the scaffold (1616 nts) in three different combinations using the aforementioned types of modified dNTPs at various ratios and performed agarose gel electrophoresis to assess the yield (Table S10 and Figure S17a,b). Scaffolds with two modifications included 5, 10, or 20% for each NH_2_ and αThiol, namely, samples multi-I, multi-II, and multi-III, respectively. The reaction efficiency based on the band intensities was similar for each modified scaffold (Figure S17a). Scaffolds with three modifications were also described in Table S10, namely, multi-IV, multi-V, and multi-VI. We performed yield quantification only for the scaffolds with three modifications after gel extraction (Figure S17c). Among the ratios of modifications, adding 10% biotin, 20% NH_2_, and 20% αThiol modified dNTPs into the reaction reduced the amount of scaffold synthesized. We obtained 0.65 pmol of unmodified scaffold, 0.66 pmol of multi-IV scaffold, and 0.89 pmol of multi-V scaffold per aPCR. We did not observe statistically significant differences among the unmodified, multi-IV, and multi-V scaffolds, but for the multi-VI scaffold, the yield decreased to 0.29 pmol per aPCR, which was 56% less than unmodified scaffold. This result demonstrated the negative effect of increased modifications on the synthesis of scaffolds.

We folded PB NPs with two of the multifunctional scaffolds (multi-IV and multi-V, Table S11) and validated the proper folding with AFM (Figure 3c and Figure S18). We checked the accessibility and binding properties of biotinylated sites of DNA-NPs by coupling them with streptavidin following the same procedure used when modifying scaffold with only biotin groups. The band shift in the gel electrophoresis (Figure S19) and the SPR experiments (Figure 3b and Figure S20) validated the attachment of DNA-NPs on streptavidin for DNA-NPs folded with biotinylated scaffolds or with biotinylated staples. DNA-NPs without any modification (no modified scaffold or modified staples) that were used as a negative control do not show bound streptavidin after injection (Figure 3b). Indeed, because of the nonspecific interaction between surface and DNA-NPs, a signal increase was observed after the initial injection for the negative control, but the signal went back to zero after ∼400 s once the dissociation step started as opposed to the biotinylated DNA-NPs (Figure 3b and Figure S13). The same samples were used for the stability assessment, along with NPs having the αThiol-only modification. The multifunctional NPs with 15% αThiol modification (multi-V) also showed an improved stability comparable to NPs folded with the 20% αThiol scaffold, with 60% of NPs still intact at the end of 10 h (Figure 3d). This result demonstrated the capability of our strategy to synthesize multifunctional scaffolds and showed that aPCR is one potential method to enrich the functionality of scaffolds by incorporating multiple dNTPs simultaneously.

#### 3.3.4 Effect of Staple Strands Stoichiometry on Functional Dna-Np Assembly

**Figure 4:**
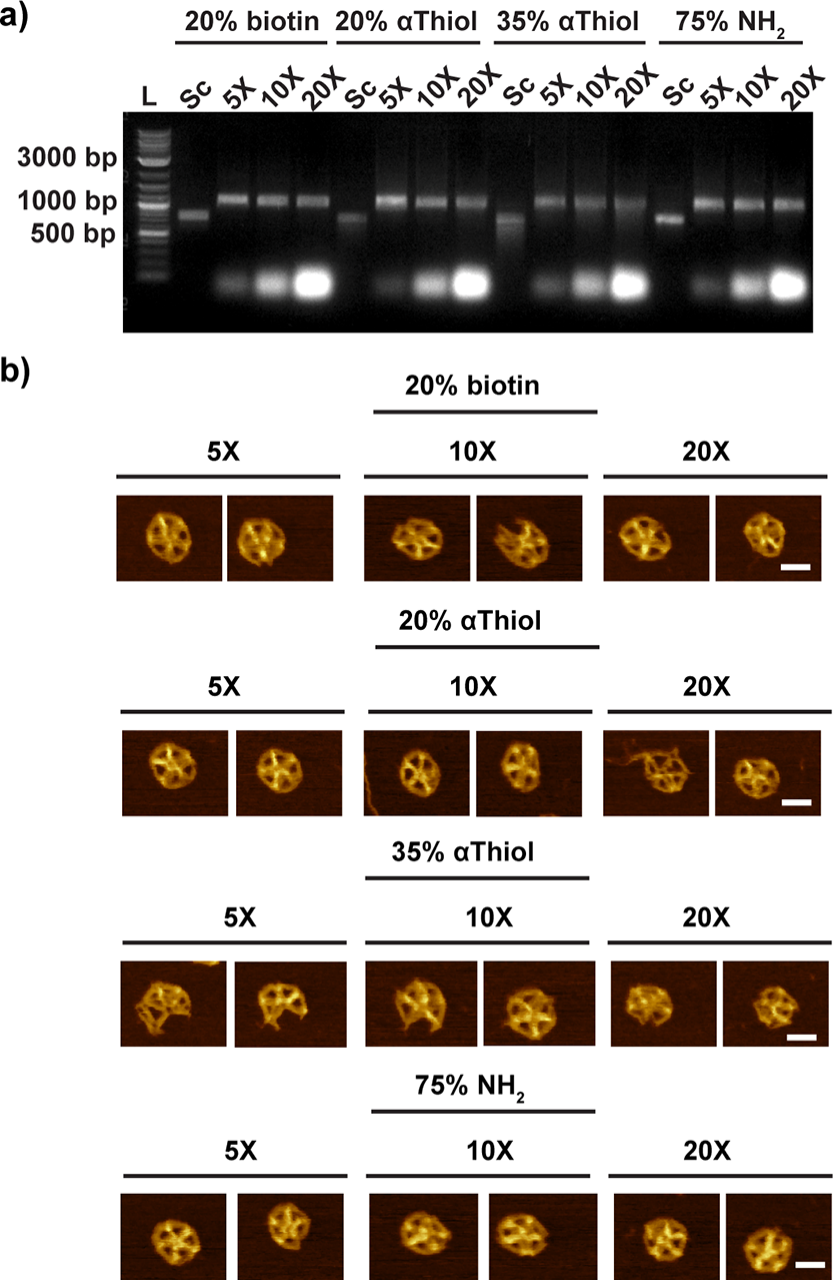
Assessing the folding of modified scaffolds with various molar ratios of staple strands. (a) Representative gel electrophoresis showing scaffolds produced with various concentrations of modified dNTPs (20% biotin, 20% αThiol, 35% αThiol, and 75% NH_2_) and DNA-NPs folded with these scaffolds and different molar concentrations of staple strands (5, 10, and 20×) and without purification. (b) Representative AFM images for the conditions tested in panel a after purification to remove the excess of staple strands (scale bar: 20 nm).

All the results from folded NPs (with or without modified scaffold) presented here were obtained using 10× excess molar ratio of staple strands as commonly used in many DNA origami studies. To evaluate how the folding efficiency is altered when using lesser and higher ratios of staple strands than 10×, we tested the assembly of DNA-NPs using 1616 nts scaffolds produced with different percentages of modified dNTPs. We selected scaffolds produced with 20% biotin-dCTPs, 20% αThiol (all dNTPs), 35% αThiol (all dNTPs), and 75% NH_2_-dCTPs and folded them with 5, 10, and 20× molar ratios of staple strand mixes. The gel image from folded particles shows that the three molar ratios yield the same folding efficiency, and no noticeable differences were observed under AFM (Figure 4a,b and Figure S16). Thus, we continued to use the usual 10× molar ratio of staple strands for all other experiments.

### 3.4 Precise Functionalization

**Figure 5:**
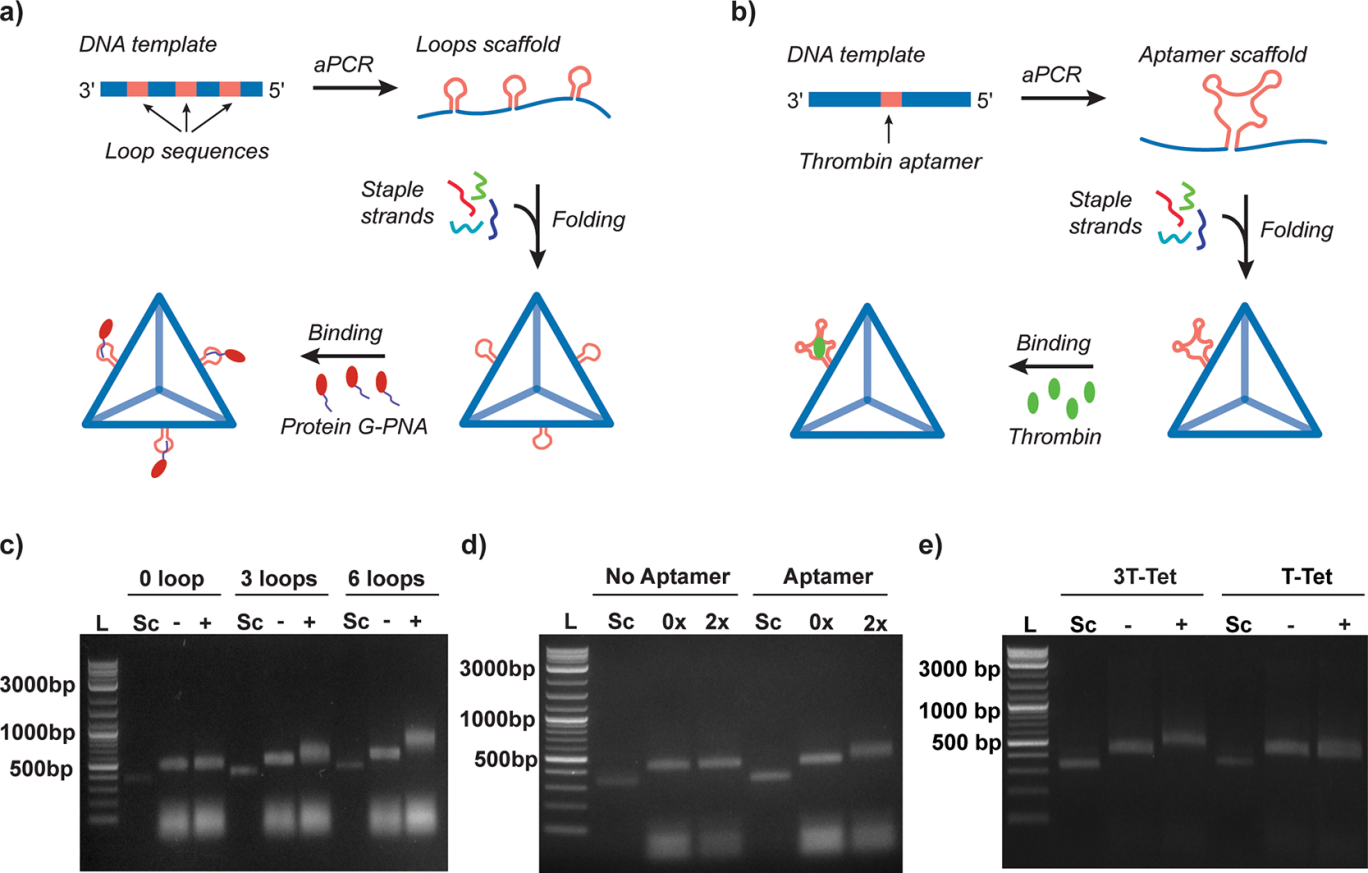
Site-specific modification of DNA origami using aPCR produced scaffolds. (a) Schematic of the aPCR-based procedures to synthetize DNA origami scaffolds with sequence-specific loops for conjugation of nucleic acid modified biomolecules (i.e., PG-PNA). (b) Schematic of the aPCR-based procedures to synthetize DNA origami scaffolds with aptamer sequences. (c) PG-PNA binding on DNA origami tetrahedra folded with scaffolds containing zero, three, and six binding loops (L: ladder; Sc: scaffold; −: without PG-PNA; and +: with PG-PNA). (d) Thrombin binding to aptamer sequence displayed on the DNA tetrahedra folded with scaffold with or without thrombin aptamer in the presence of 0 and 2× molar ratio of thrombin. (e) Thrombin binding to three aptamer sequences displayed on the DNA tetrahedra folded. All gel images in the three panels show the purified scaffolds and their corresponding unpurified NPs with or without protein modifications (L: ladder; Sc: scaffold; 3 T-Tet: tetrahedron with three 15-mer thrombin aptamer sequences; T-Tet: tetrahedron with one 29 nts thrombin aptamer sequence; −: without thrombin; and +: with thrombin).

One of the main advantages of using aPCR over bacteriophage production is the control over the sequence in terms of base composition and length. Leveraging this advantage and using a DNA origami designing software, we designed scaffolds that include specific sequences that will not participate in the assembly of the NP but instead form stem loops that are precisely located on the folded NPs (Figure 5a,b). Carefully designing the loop sequence allows further attachment of biomolecules tagged with complementary strands via simple hybridization similar to using staple strands with ssDNA overhangs. However, whereas the overhangs on staples must be placed at the 5′- or 3′-ends of the staple strands, here the loop can be placed with less constraints on the scaffold or in addition to overhangs displayed by staple strands to increase the number of binding sites. This would also eliminate the need to purify modified staple strands after folding and before hybridization of the biomolecules, which would reduce the loss of NPs in subsequent purification steps. To generate the sequence specific templates required to amplify ssDNA, we used custom gBlocks, which are in general lower than 3 kb long synthetic double-stranded DNA fragments synthesized *de novo*. Although using small synthetic dsDNA fragments certainly constrains the size of the DNA-NPs that can be folded because of their length limitations, our strategy can be easily applicable to plasmid construction via common cloning methods or by using custom plasmid synthesis that can be designed with length longer than 3 kb.

#### 3.4.1 DNA Loops for Protein Binding via Peptide Nucleic Acid Hybridization

A scaffold sequence (522 nts long) was modified to display different numbers of 9 nts long loops (one, three, or six) for binding (Figure 5a) on a tetrahedral structure, which also included a flanking loop stem and variable regions of 27 nts in length. It is important to note that optimizing the sequence design of the loops is critical for the viable synthesis of the gBlocks. Linear gene synthesis has some limitations that preclude the use of long repeated sequences, which forced us to design the loop with a constant region (binding region) and one with a variable region that changed for each loop (Table S7). Loops on the scaffold serve as anchor sites for the hybridization of complementary nucleic acids. Here, we designed the binding sequence to be complementary to a peptide nucleic acid (PNA) strand as this method allows the use of a shorter linker due to the higher affinity of PNA for DNA rather than DNA itself. We chose a recombinant protein G (PG) commonly used to bind and immobilize antibodies via their Fc domains.^23^ The PG has a free cysteine at its N-terminal that we used to conjugate with a PNA maleimide to form protein G-PNA (PG-PNA) as recently described.^23^ Formations of DNA tetrahedron with zero, one, three, or six loops and further binding of PG-PNA to DNA tetrahedron NPs were examined with agarose gel electrophoresis without any purification steps (Figure 5c and Figure S21). Folding was confirmed with gel electrophoresis. Another shift was observed when using PG-PNA with three and six loops but not with zero loop. The binding with one loop also did not provide the clear shift in gel electrophoresis (Figure S21). Our results demonstrate that we achieved insertion of the loop sequences into the scaffold without hampering the folding of the NP and that the loops are accessible for hybridization of nucleic acid labeled biomolecules. Additionally, we compared the binding efficiency of DNA-NPs folded with modified scaffolds and with modified staples for functionalization with protein G. The gel image (Figure S22) shows a larger shift for protein-bound DNA-NPs that were folded with modified staples and purified in comparison to NPs with modified scaffolds that were not purified. This result might be due to the limited accessibility to the binding region of the loop vs using free ssDNA overhangs on the staple. This specific design point will be the subject of a future study.

#### 3.4.2 DNA Loops for Aptamer Functionalization

We further introduced aptamer sequences into the scaffold (Figure 5b and Table S10). Aptamers are short single-stranded DNA or RNA molecules selected among a vast library of oligonucleotides to have a strong affinity for binding to a desired biomolecule via target-specific shape change.^58^ Given that they are nucleic acid in nature, they can be directly inserted into the DNA scaffold sequence without the need for additional chemistry as needed for antibodies and can be used for selective recognition and binding to target molecules with high affinity.^59^

Here, we incorporated a well-studied and characterized 29-nts DNA thrombin aptamer into the sequence of a scaffold (522 nts long) to assemble a tetrahedron DNA-NP.^60^ One aptamer sequence incorporation was performed during the gBlock design stage as for the loops presented in the previous section. The agarose gel in Figure 5d shows the comparison of DNA scaffolds and their folded NPs with and without thrombin binding. The size change induced a shift for the folded NP, with and without the aptamer present, in the absence of thrombin or with 2× molar excess of thrombin. The gel validated the binding of thrombin with a clear shift, which confirmed that the aptamers were properly folded on the NPs and retained the capacity of binding. The gel characterization of gBlock-derived DNA-NP functionalization with protein G and thrombin shows a clear shift. However, because of the size of the NPs, AFM images do not allow us to confirm the binding of proteins to DNA-NPs using AFM (Figure S23).

These results demonstrate the precise functionalization of DNA-NPs via scaffold sequence design, specifically by inserting aptamer sequences, and the potential of our strategy. As for the loop design, for long aptamers, the gBlocks strategy will not allow the user to add more than one aptamer with the same sequence if the sequence length is too long. Therefore, use of shorter aptamer sequences or custom plasmid synthesis might be more appropriate to display multiple long aptamer sequences. For instance, using a 15-mer thrombin aptamer, we were able to design gBlocks with three aptamers displayed on their surface that can bind thrombin efficiently (Figure 5e and Table S10).

Given the potential of functionalized DNA-NPs to be used *in vivo*, the presence of endotoxin needs to be checked to ensure their safety. To assess the biocompatibility of the scaffolds and the folded DNA-NPs, we tested the presence of endotoxin after purification of the 1644 nts scaffold and after folding of the same scaffold into 6-HB.The initial endotoxin level measured was 1.98 ± 0.89 EU/mL (*n* = 3) for 10 nM of the produced 1644 nts scaffold as measured with the ToxinSensor Chromogenic LAL Endotoxin Assay Kit (Figure S24). After folding and purifying the 6-HB NPs tested at 10 nM using our synthetized scaffold, the level of endotoxin was found to be about 0.11 ± 0.01 EU/mL (*n* = 3). This result demonstrated that scaffolds produced with aPCR have a low level of endotoxin and that the purification of NPs from excess staples via centrifugal filtration reduces the level of endotoxin from the folded NPs to make them compatible with biomedical applications.

## 4 Conclusions

The emergence of several design and synthesis methods that facilitated the synthesis of DNA origami has led to a broader use of functional DNA origami for biomedical applications such as drug delivery, bioimaging, cancer immunotherapy, and vaccine delivery. However, the common method of modification still relies on modified staples for functionalizing the DNA-NPs. Because of the high cost of modified staples and the extra purification steps required for the removal of excess oligonucleotides after folding, prefunctionalization of the scaffold could provide a significant advantage. While allowing the production of large amount of scaffold, bacteriophage production does not allow for direct modification of scaffolds other than tuning the sequence and the sequence length. On the other hand, PCR-based methods provide alternatives for ssDNA production; however, they require the use of enzymatic or magnetic-bead separation of ssDNA from the dsDNA, which affects the cost of the synthesis process and yield of the ssDNA product obtained. Even though there are a few techniques available to produce modified scaffolds, aPCR is currently one of the best alternative methods in its ability to readily and rapidly produce modified ssDNA scaffolds with custom lengths and sequences and incorporation of modified dNTPs. Importantly, different purification techniques (e.g., chromatography) should be also tested to increase the yield of ssDNA scaffolds and scale up their production because gel purification is hard to scale up.

In this study, we demonstrated that aPCR can be used to synthesize DNA origami scaffolds with a variety of modifications, including site-specific loops and aptamers for precise binding of biomolecules. Scaffolds with different lengths of nucleotides were easily modified using commercially available modified dNTPs. The aPCR approach is versatile in the kind of template DNA strand that can be used; in this work, we show the application of gBlocks and m13mp18 ssDNA as templates. While gBlocks are ideal for custom sequences of 500–3000 bp that could even encode proteins for gene expression, current solid-state DNA synthesis paradigms do not allow complete access to an arbitrary sequence space. Much like traditional PCR, dsDNA plasmids are expected to be just as compatible with aPCR for scaffold synthesis as shown with the lambda phage in this study. This simple and efficient method is preferable to alternatives that have scaffold size restrictions and require more time and cost. Regardless of scaffold size, our technique can produce ssDNA for less than $5/pmol depending on enzyme selection. Furthermore, because it is produced entirely in-house, it is not subject to the same susceptibilities of inventory issues and turnaround time. Incorporation efficiency of either single or multiple functional groups into the scaffold confirmed previous findings that increasing the number of modifications can decrease the yield of scaffold synthesized. Each replacement of functional nucleotides on ssDNA scaffolds in aPCR was confirmed via different types of characterization methods. With this, we were able to assess the way to increase the stability of nanoparticles without coating with any biomolecules or preparing for further functionalization. In addition to nonspecific modifications on the scaffold, we also were able to insert aptamer/loop sequences into the scaffold enabling position-specific functionalization. Overall, the practicality of this strategy for the synthesis of ssDNA scaffold and the ease of enrichment of scaffold functionality with nonspecific or specific functionalization in a one-pot reaction will pave the way for many applications requiring functionalized DNA origami scaffolds or DNA-NPs.

## Data Availability

All data will be available upon reasonable request to the corresponding author.

## Supporting Information Available

All scaffold sequences, the aPCR primer sequences, the staple strand sequences used to fold the DNA origami nanoparticles, and supplementary results (including agarose gel images, quantification of ssDNA production, and full AFM images, SPR graphs, and endotoxin result) (PDF)
