# Supplementary material for: Customized Scaffolds for Direct Assembly of Functionalized DNA Origami
Source: ACS Appl Mater Interfaces. 2023 Jun 2;15(23):27759–73. doi: 10.1021/acsami.3c05690 (PMC10273176; doi:10.1021/acsami.3c05690)
Supplement: Supplementary file 1 — am3c05690_si_001.pdf [file am3c05690_si_001.pdf]

Supporting Information

Customized Scaffolds for Direct Assembly of Functionalized DNA Origami

Esra Oktay [1,3†], Joshua Bush [1,3†], Merlyn Vargas [1†], Dylan Valerio Scarton [2,3], Bailey O'Shea [1], Amber Hartman [1], Christopher M. Green[4], Kayla Neyra[5], Carolina M. Gomes[1,3], Igor L. Medintz[4], Divita Mathur[5], Remi Veneziano [1,3]*.*

[1] George Mason University, College of Computing and Engineering, Department of Bioengineering, Fairfax VA, USA;

[2] George Mason University, College of Science, Interdisciplinary Program in Neuroscience, Fairfax, VA, USA

[3] Institute for Advanced Biomedical Research, Manassas VA, USA;

[4] Center for Bio/Molecular Science and Engineering Code 6900, U.S. Naval Research Laboratory, Washington DC, USA;

[5] Department of Chemistry, Case Western Reserve University, Cleveland OH, USA;

[†] Equal Contribution;

***** Correspondence: rvenezia@gmu.edu; dxm700@case.edu

Table S1. Single-strand DNA (ssDNA) scaffold sequences synthetized using M13mp18 ssDNA as template (red bases indicate the primer [forward] or primer binding sequences [reverse]).

**449 nts ssDNA scaffold sequence**
**GTCGTCGTCCCCTCAAACT**GGCAGATGCACGGTTACGATGCGCCCATCTACACCAACGTAACCTATCCCATTACGGTCAATC<br>CGCCGTTTGTTCCCACGGAGAATCCGACGGGTTGTTACTCGCTCACATTTAATGTTGATGAAAGCTGGCTACAGGAAGGCCAG<br>ACGCGAATTATTTTTGATGGCGTTCCTATTGGTTAAAAAATGAGCTGATTTAACAAAAATTTAACGCGAATTTTAACAAAATAT<br>TAACGTTTACAATTTAAATATTTGCTTATACAATCTTCCTGTTTTTGGGGCTTTTCTGATTATCAACCGGGGTACATATGATTGA<br>CATGCTAGTTTTACGATTACCGTTCATCGATTCTCTTGTTTGCTCCAGACTCTCAGGCAATGACCTGATAGCCTTTGTAGATCTC<br>TCAAAAATAG**CTACCCTCTCCGGCATTAAT**
**1,616 nts ssDNA scaffold sequence**
**GCGACGATTTACAGAAGCAA**GGTTATTCACTCACATATATTGATTTATGTACTGTTTCCATTAAAAAAGGTAATTCAAATGAA<br>ATTGTTAAATGTAATTAATTTTGTTTTCTTGATGTTTGTTTCATCATCTTCTTTTGCTCAGGTAATTGAAATGAATAATTCGCCTC<br>TGCGCGATTTTGTAACTTGGTATTCAAAGCAATCAGGCGAATCCGTTATTGTTTCTCCCGATGTAAAAGGTACTGTTACTGTAT<br>ATTCATCTGACGTTAAACCTGAAAATCTACGCAATTTCTTTATTTCTGTTTTACGTGCTAATAATTTTGATATGGTTGGTTCAAT<br>TCCTTCCATAATTCAGAAGTATAATCCAAACAATCAGGATTATATTGATGAATTGCCATCATCTGATAATCAGGAATATGATGA<br>TAATTCCGCTCCTTCTGGTGGTTTCTTTGTTCCGCAAAATGATAATGTTACTCAAACTTTTAAAATTAATAACGTTCGGGCAAAG<br>GATTTAATACGAGTTGTCGAATTGTTTGTAAAGTCTAATACTTCTAAATCCTCAAATGTATTATCTATTGACGGCTCTAATCTAT<br>TAGTTGTTAGTGCACCTAAAGATATTTTAGATAACCTTCCTCAATTCCTTTCTACTGTTGATTTGCCAACTGACCAGATATTGAT<br>TGAGGGTTTGATATTTGAGGTTCAGCAAGGTGATGCTTTAGATTTTTCATTTGCTGCTGGCTCTCAGCGTGGCACTGTTGCAGG<br>CGGTGTTAATACTGACCGCCTCACCTCTGTTTTATCTTCTGCTGGTGGTTCGTTCGGTATTTTTAATGGCGATGTTTTAGGGCTA<br>TCAGTTCGCGCATTAAAGACTAATAGCCATTCAAAAATATTGTCTGTGCCACGTATTCTTACGCTTTCAGGTCAGAAGGGTTCT<br>ATCTCTGTTGGCCAGAATGTCCCTTTTATTACTGGTCGTGTGACTGGTGAATCTGCCAATGTAAATAATCCATTTCAGACGATT<br>GAGCGTCAAAATGTAGGTATTTCCATGAGCGTTTTTCCTGTTGCAATGGCTGGCGGTAATATTGTTCTGGATATTACCAGCAAG<br>GCCGATAGTTTGAGTTCTTCTACTCAGGCAAGTGATGTTATTACTAATCAAAGAAGTATTGCTACAACGGTTAATTTGCGTGAT<br>GGACAGACTCTTTTACTCGGTGGCCTCACTGATTATAAAAACACTTCTCAAGATTCTGGCGTACCGTTCCTGTCTAAAATCCCT<br>TTAATCGGCCTCCTGTTTAGCTCCCGCTCTGATTCCAACGAGGAAAGCACGTTATACGTGCTCGTCAAAGCAACCATAGTACGC<br>GCCCTGTAGCGGCGCATTAAGCGCGGCGGGTGTGGTGGTTACGCGCAGCGTGACCGCTACACTTGCCAGCGCCCTAGCGCCCG<br>CTCCTTTCGCTTTCTTCCCTTCCTTTCTCGCCACGTTCGCCGGCTTTCCCCGTCAAGCTCTAAATCGGGGGCTCCCTTTAGGGTTC<br>CGATTTAGTGCTTTACGGCACCTCGACCCCAAAAAACTTGATTTGGGTGATGGTTCACGTAGTGGGCCATCGCCCTG**ATAGAC**<br>**GGTTTTTCGCCCTT**
**1,644 nts ssDNA scaffold sequence**
**CCCTTTAGGGTTCCGATTTA**GTGCTTTACGGCACCTCGACCCCAAAAAACTTGATTTGGGTGATGGTTCACGTAGTGGGCCAT<br>CGCCCTGATAGACGGTTTTTCGCCCTTTGACGTTGGAGTCCACGTTCTTTAATAGTGGACTCTTGTTCCAAACTGGAACAACAC<br>TCAACCCTATCTCGGGCTATTCTTTTGATTTATAAGGGATTTTGCCGATTTCGGAACCACCATCAAACAGGATTTTCGCCTGCTG<br>GGGCAAACCAGCGTGGACCGCTTGCTGCAACTCTCTCAGGGCCAGGCGGTGAAGGGCAATCAGCTGTTGCCCGTCTCACTGGT<br>GAAAAGAAAAACCACCCTGGCGCCCAATACGCAAACCGCCTCTCCCCGCGCGTTGGCCGATTCATTAATGCAGCTGGCACGAC<br>AGGTTTCCCGACTGGAAAGCGGGCAGTGAGCGCAACGCAATTAATGTGAGTTAGCTCACTCATTAGGCACCCCAGGCTTTACA<br>CTTTATGCTTCCGGCTCGTATGTTGTGTGGAATTGTGAGCGGATAACAATTTCACACAGGAAACAGCTATGACCATGATTACGA<br>ATTCGAGCTCGGTACCCGGGGATCCTCTAGAGTCGACCTGCAGGCATGCAAGCTTGGCACTGGCCGTCGTTTTACAACGTCGT<br>GACTGGGAAAACCCTGGCGTTACCCAACTTAATCGCCTTGCAGCACATCCCCCTTTCGCCAGCTGGCGTAATAGCGAAGAGGC<br>CCGCACCGATCGCCCTTCCCAACAGTTGCGCAGCCTGAATGGCGAATGGCGCTTTGCCTGGTTTCCGGCACCAGAAGCGGTGC<br>CGGAAAGCTGGCTGGAGTGCGATCTTCCTGAGGCCGATACTGTCGTCGTCCCCTCAAACTGGCAGATGCACGGTTACGATGCG<br>CCCATCTACACCAACGTGACCTATCCCATTACGGTCAATCCGCCGTTTGTTCCCACGGAGAATCCGACGGGTTGTTACTCGCTC<br>ACATTTAATGTTGATGAAAGCTGGCTACAGGAAGGCCAGACGCGAATTATTTTTGATGGCGTTCCTATTGGTTAAAAAATGAG<br>CTGATTTAACAAAAATTTAATGCGAATTTTAACAAAATATTAACGTTTACAATTTAAATATTTGCTTATACAATCTTCCTGTTTT<br>TGGGGCTTTTCTGATTATCAACCGGGGTACATATGATTGACATGCTAGTTTTACGATTACCGTTCATCGATTCTCTTGTTTGCTC<br>CAGACTCTCAGGCAATGACCTGATAGCCTTTGTAGATCTCTCAAAAATAGCTACCCTCTCCGGCATTAATTTATCAGCTAGAAC<br>GGTTGAATATCATATTGATGGTGATTTGACTGTCTCCGGCCTTTCTCACCCTTTTGAATCTTTACCTACACATTACTCAGGCATT<br>GCATTTAAAATATATGAGGGTTCTAAAAATTTTTATCCTTGCGTTGAAATAAAGGCTTCTCCCGCAAAAGTATTACAGGGTCAT<br>AATGTTTTTGGTACAACCGATTTAGCTTTATGCTCTGAGGCTTTATTGCTTAATTTTGCTAATTCTTTGCCTTGCCTGTATGATTT<br>ATTGGATGTTAATGCTACTACTATTAGTAGA**ATTGATGCCACCTTTTCAGC**

Table S2. Primer sets for amplification of ssDNA scaffolds by asymmetric PCR (aPCR).

449 nts forward primer	GTCGTCGTCCCCTCAAACT
449 nts reverse primer	ATTAATGCCGGAGAGGGTAG
All gBlocks^{tm} forward primer	GGACGCTATCCAGTCTAAACAT
All gBlocks^{tm} reverse primer	GAAAGAGGACAGATGAACGGTG
1,616 nts forward primer	GCGACGATTTACAGAAGCAA
1,616 nts reverse primer	AAGGGCGAAAAACCGTCTAT
1,644 nts forward primer	CCCTTTAGGGTTCCGATTTA
1,644 nts reverse primer	GCTGAAAAGGTGGCATCAAT

Table S3. Unmodified staple strand sequences for folding 1,616 nucleotides (nts) and 1,644 nts scaffolds into pentagonal bipyramid (PB) and six-helix bundle (6HB) DNA nanoparticles (DNA-NPs), respectively.

PB staples	Unmodified sequence
Staple-2	CCACCGAGTAATTTTTAAGAGTCTGTTCTTTGATTAGTTTTTAATAACATC
Staple-3	GGCCTTGCTGGTTTTTTAATATCCAGTAGACAGGAACTTTTTGGTACGCCAG
Staple-4	GAAGGTTATCTTTTTTAAAATATCTTCGTCAATAGATTTTTTAATACATTTG
Staple-5	ATATAATCGTTGGCAAATCAACAGTAGAAAG
Staple-6	GAATTGAGTATCAGATGATGGCAATTCATCA
Staple-7	CGCTGGCAAAGCGAAAGGAGCGGGTATTAAT
Staple-8	TTTAAAAGCCTTTGCCCGAACGTCGCTAGGG
Staple-9	GCAATACTCCATCACGCAAATTAATAGACTT
Staple-10	TACAAACAAGGATTTAGAAGTATCCGTTGTA
Staple-11	ATTCGACAACTTTTTTCGTATTAAATTTTGAGTAACATTTTTTTATCATTTTAATTATCATCATTTTTTATTCCT GAT
Staple-12	AGGAGCGGGCGGAACAAAGAAACATGATGAA
Staple-13	ACAAACATACCTGAGCAAAAGAAGCACCAGA
Staple-14	ATTAGAGCTAGGTGCACTAACAATAAGAATA
Staple-15	CGTGGCACTTCTGACCTGAAAGCGCTAATAG
Staple-16	GAGAGCCAAACAGAAATAAAGAAATTGCGTA
Staple-17	GATTTTCAACCGCCTGCAACAGTGCCACGCT
Staple-18	GGGAGAAACAATATATGTGAGTGGGCCCACT
Staple-19	ACGTGAACACAGTAACAGTACCTTTTACATC
Staple-20	GGTTTAACGTCTTTTTAGATGAATATCATCACCCAAATTTTTTCAAGTTTTT
Staple-21	TCCTCGTTGGATTTTTATCAGAGCGGAAAACGCTCATTTTTTGGAAATACCTGGTGAGGCGGTTTTTTCAGT ATTAA
Staple-22	ACGTGCTTTGGGGTCGAGGTGCCGTAAAGCA
Staple-23	CTAAATCGGGTTGCTTTGACGAGCACGTATA
Staple-24	AAAACAGAACATTTTGACGCTCAATCGTCTG
Staple-25	AAATGGATCGAACGAACCACCAGCAGAAGAT
Staple-26	TGGCGAGAAAGTTTTTGAAGGGAAGAAGTGTAGCGGTTTTTTCACGCTGCGC
Staple-27	CAGTGAGGAATCTTGAGAAGTGTTGCCGCGC
Staple-28	CCGCTACAGGGTTTTTCGCGTACTATGAACCCTAAAGTTTTTGGAGCCCCCG
Staple-29	TTAATGCGGTAACCACCACACCCTTTATAAT
Staple-30	CCCTCAATCAATTTTTTATCTGGTCACTGATTGTTTGTTTTTGATTATACTT
Staple-31	ATCAAAATTATTTTTTTAGCACGTAAGCAGCAAATGATTTTTAAAATCTAAA
Staple-32	CGCGCAGAGCTTTGAATACCAAGTAATTGAA
Staple-33	CCAACCATCTGAATTATGGAAGGTACAAAAT
Staple-34	TAGCCCTAATTAGTCTTTAATGCGACCTCAA
Staple-35	ATATCAAAGCATCACCTTGCTGACGAACTGA

Staple-36	TATTTACATTGTTTTTGCAGATTCACTGGCCAACAGATTTTTGATAGAACCC
Staple-37	AGACAATATTTTTTTTTGAATGGCTAAACATCGCCATTTTTTTAAAAATAC
Staple-38	AAACTATCACTTGCCTGAGTAGAAATAAAAG
Staple-39	GGACATTCCAGTCACACGACCAGTAGAACTC
Staple-40	GGCGAATTATTTTTTTCATTTCAATTCAAGAAAACAATTTTTAATTAATTAC
Staple-41	TAATGGAAACATTTTTGTACATAAATCAATAACGGATTTTTTTCGCCTGATT
Staple-42	GGCGAACGATTTAGAGCTTGACGGTTGAATT
Staple-43	ACCTTTTTATTTAACAATTTCATGGAAAGCC
Staple-44	AGGGATTTAACAATATTACCGCCAGCCATTG
Staple-45	CAACAGGAGAGCTAAACAGGAGGCCGATTAA
**6HB staples**	**Unmodified sequence**
Staple-1	TGCTGCATTCACCACATTAAT
Staple-2	AATTCGTTGTTATCTTTTGTTAAATCAGCTCAT
Staple-3	GCGGGCCTGGCCCTTCACTGC
Staple-4	AAGATCGAAGAATAAATGTGT
Staple-5	TTGACCGGTCAATCTGAGAGA
Staple-6	CCATCAAATTAAATCGCTCAC
Staple-7	TCGTAACGTAAACAGGAGCTG
Staple-8	GAGGCGGCACAACATTGTTAAAATTCGCAAATAATTCGCGTC
Staple-9	ATCACCAAGAGTCTAGAGAATCGATGAATGTAGATGGGCGCA
Staple-10	AGGTAAATTTTGCGTTTTGGGG
Staple-11	TTTTTAACCAATAAACGAC
Staple-12	CCAGCTGAGCTGATGGAAACC
Staple-13	TGGCCTTTAATATTTACGAGC
Staple-14	CTGTTGGAAGCGGTATAAATT
Staple-15	TCCGTGGTGATAATTTGCGTT
Staple-16	AGAAAGGAACATTAAAGCAGCTAAATCGCGTGCATCTGGTGC
Staple-17	TTGTTTCCTGT
Staple-18	AATTCCATTGTACCCCCGGTGCGTATTGCCAGGGTTTTCCCA
Staple-19	TTTGAGGTGCCGTATGACCCT
Staple-20	TTGCCTGTCAATATGGCGAAAATCCTGTCAGGCAAAGCGCCA
Staple-21	TGCAATGTTCAACGAACCATCACCCAAACAGTGGAAGAACGG
Staple-22	AATGCCGCTATTTTATATGTACCCCGGTGAACAAACGGCGGA
Staple-23	CTAACTCCCAGTCGTGCCCTTCACCGCCTCTTCGCCGGATTC
Staple-24	CGGAAGCCCAACGCGGGTGGTTTTTCTTAGGCGATCCAGCTT
Staple-25	GAGTAACAAACAGGGAGTGAG
Staple-26	GTCACGAGAGGATCGAGCTCG
Staple-27	TCAGGGCGATGGCCCACTACGTGCAAGGATAAAAATTTTTAG
Staple-28	TCATCAAAAATATTGTAAAGC
Staple-29	TTCGCCACCCAGCAGATATTC
Staple-30	TCGGCCTTCAAGTTGGAGAAG

Staple-31	CGGAAACTTGATGGCAGTCAA
Staple-32	GGTAACGGGCGCCAGCGGGGA
Staple-33	GTCAAAGGGCGAAAAACCGTCTA
Staple-34	AACCGTTAGGCTATCGTAAAACTAGCATTAATGGGTGCGCAA
Staple-35	TTGGAACAAGAGTCCATGAGTGTTGTTCCAGT
Staple-36	TCTACAACTAGCTGCCACGCTGGTTTGCTTCAGGCATAGGTC
Staple-37	GAATCGGATAAAGTTAAATTGTAAACGTCCTGTAGTAAGTTG
Staple-38	GGCCAGTGCCAA
Staple-39	GCGGTAGGAGAGGCGAGAGAGTTGCAGCGAAGGGCGATCGGT
Staple-40	TCGAGGGGACGACGACAGTA
Staple-41	GCTTGCATGCCTGTCATAGCT
Staple-42	CCTTTATCCTGAGTGCCCGAGATAGGGTCTATTAACTCCAAC
Staple-43	ACGTTGGCGGTAATCAGGTCA
Staple-44	TTCCGGCGCAAAATAAGGGTG
Staple-45	TACCAAACCGGAGATGGTTCCGAAATCGACCGCTTCTGCCAG
Staple-46	CCGCTTTACATTAACAGAAAAGCCCCAAAACCCGTTATTACG
Staple-47	CTGGGGTCCAGCTGGTGAGACGGGCAACGCGAAAGGGGGATG
Staple-48	GTGAAATAATCATGGCAGGTCGACTCTACGTTGTAAGGAACG
Staple-49	AACCCTCATATATTTTAAA
Staple-50	GTAATACGATTCAACCCTTATAAATCAACACTCCAGCCAGCT
Staple-51	TGTCGTGGCCTAATAAGATTGTATAAGCCATTAAATGTGAGC

Table S4. Dye modified-staple strands for Förster resonance energy transfer (FRET) assay with PB.

Name	Sequence
Fret1-Acc1-PB10	TAMRA/TACAAACAAGGATTTAGAAGTATCCGTTGTA
Fret1-Don1-PB11	FAM/ATTCGACAACTTTTTTCGTATTAAATTTTGAGTAACATTTTTTTATCATTTTAATTATCATCATTTTTATTCCTGAT
Fret2-Don2-PB21	FAM/TCCTCGTTGGATTTTTATCAGAGCGGAAAACGCTCATTTTTTGGAAATACCTGGTGAGGCGGTTTTTTCAGTATTAAC
Fret2-Acc2-PB22	TAMRA/ACGTGCTTTGGGGTCGAGGTGCCGTAAAGCA

Table S5. Biotinylated staple strands of 6-HB.

6-HB staples	Biotinylated sequence
2[112]	GCG GGC CTG GCC CTT CAC TGC
3[181]	TCG TAA CGT AAA CAG GAG CTG
3[97]	GAG TAA CAA ACA GGG AGT GAG
2[28]	GTC ACG AGA GGA TCG AGC TCG
2[196]	TTC CGG CGC AAA ATA AGG GTG

Table S6. Biotinylated staple strands of PB.

PB staples	Biotinylated sequence
Staple-17	/5Biosg/GAT TTT CAA CCG CCT GCA ACA GTG CCA CGC T
Staple-19	/5Biosg/ACG TGA ACA CAG TAA CAG TAC CTT TTA CAT C
Staple-22	/5Biosg/ACG TGC TTT GGG GTC GAG GTG CCG TAA AGC A
Staple-24	/5Biosg/AAA ACA GAA CAT TTT GAC GCT CAA TCG TCT G
Staple-45	/5Biosg/CAA CAG GAG AGC TAA ACA GGA GGC CGA TTA A

Table S7. All gBlocks™ sequences used to fold the tetrahedron 42-bp edge length without loop and with one, three, and six-loop, respectively. (Red bases represent the primer [forward] and primer binding sites [reverse], orange bases represent the stem of the loop, blue bases represent the binding region of the loop, and the bolded black bases represent the variable sequence of the loop).

**gBlocks™ sequence without loop**
**GGACGCTATCCAGTCTAAACAT**TTTACTATTACCCCCTCTGGCAAAACTTCTTTTGCAAAAGCCTCTCGCTATTTTGGTTTTTA<br>TCGTCGTCTGGTAAACGAGGGTTATGATAGTGTTGCTCTTACTATGCCTCGTAATTCCTTTTGGCGTTATGTATCTGCATTAGTT<br>GAATGTGGTATTCCTAAATCTCAACTGATGAATCTTTCTACCTGTAATAATGTTGTTCCGTTAGTTCGTTTTATTAACGTAGATT<br>TTTCTTCCCAACGTCCTGACTGGTATAATGAGCCAGTTCTTAAAATCGCATAAGGTAATTCACAATGATTAAAGTTGAAATTAA<br>ACCATCTCAAGCCCAATTTACTACTCGTTCTGGTGTTTCTCGTCAGGGCAAGCCTTATTCACTGAATGAGCAGCTTTGTTACGTT<br>GATTTGGGTAATGAATATCCGGTTCTTGTCAAGATTACTCTTGATGAAGGTCAGCCAGCCTATGCGCCTGGTCTGTA**CACCGTT**<br>**CATCTGTCCTCTTTC**
**gBlocks™ sequence with 1-loop**
**GGACGCTATCCAGTCTAAACAT**TTTACTATTACCCCCTCTGGCAAAACTTCTTTTGCAAAAGCCTCTCGCTATTTTGGTTTTTA<br>TCGTCGTCTGGTAAACGAGGGTTATGATAGTGTTGCTCTTACTATGCCTCGTAATTCCTTTTGGCGTTATGTATCTGCATTAGTT<br>GAATGTGGTATTCCTAAATCTCAACTGATGAATCTTTCTACCTGTAATAATGTTGTTCCGTTAGTTCGTTTTATTAACGTAGATT<br>TTTCTTCCCAACGTCCTGACTGGTATAATGAGCCAGTTCTTAA**TTCGAAACTGGACTGCCGGACGCGCTGCCATTCGTT**AAT<br>CGCATAAGGTAATTCACAATGATTAAAGTTGAAATTAAACCATCTCAAGCCCAATTTACTACTCGTTCTGGTGTTTCTCGTCAG<br>GGCAAGCCTTATTCACTGAATGAGCAGCTTTGTTACGTTGATTTGGGTAATGAATATCCGGTTCTTGTCAAGATTACTCTTGAT<br>GAAGGTCAGCCAGCCTATGCGCCTGGTCTGTA**CACCGTTCATCTGTCCTCTTTC**
**gBlocks™ sequence with 3-loop**
**GGACGCTATCCAGTCTAAACAT**TTTACTATTACCCCCTCTGGCAAAACTTCTTTTGCAAAAGCCTCTCGCTATTTTGGTTTTTA<br>TCGTCGTCTGGTAAACGAGGGTTATGATAGTGTTGCTCTTACTATGCCTCGTAATTCCTTTTGGCGTTATGTATCTGCATTAGTT<br>GAATGTGGTATTCCTAAATCTCAACTGATGAATCTTTCTACCTGTAATAATGTTGTTCCGTTAGTTCGTTTTATTAACGTAGATT<br>T**TTCGATACTGGACTGTGGAGCGCCGTCCGGATCGTT**TTCTTCCCAACGTCCTGACTGGTATAATGAGCCAGTTCTTAA**TTC**<br>**GAAACTGGACTGCCGGACGCGCTGCCATTCGTT**AATCGCATAAGGTAATTCACAATGATTAAAGTTGAAATTAAA**TTCGTT**<br>**ACTGGACTGCCGACGCCCGTGACGAACGTT**CCATCTCAAGCCCAATTTACTACTCGTTCTGGTGTTTCTCGTCAGGGCAAGC<br>CTTATTCACTGAATGAGCAGCTTTGTTACGTTGATTTGGGTAATGAATATCCGGTTCTTGTCAAGATTACTCTTGATGAAGGTC<br>AGCCAGCCTATGCGCCTGGTCTGTA**CACCGTTCATCTGTCCTCTTTC**
**gBlocks™ sequence with 6-loop**
**GGACGCTATCCAGTCTAAACAT**TTTACTATTACCCCCTCTGGCAAAACTTCTTTTGCAAAAGCCTCTCGCTATTTTGGTTTTTA<br>TCGTCGTCTGGTAAACGAGGGTTA**TTCGAGACTGGACTGTGGGACGTCGGCCCACTCGTT**TGATAGTGTTGCTCTTACTAT<br>GCCTCGTAATTCCTTTTGGCGTTATGTATCTGCATTAGTTGAATGTGGTATTCCTAAATCTCA**TTCGACACTGGACTGCTGTCA**<br>**CGCCGGGCCGTCGTT**ACTGATGAATCTTTCTACCTGTAATAATGTTGTTCCGTTAGTTCGTTTTATTAACGTAGATTT**TTCGAT**<br>**ACTGGACTGTGGAGCGCCGTCCGGATCGTT**TTCTTCCCAACGTCCTGACTGGTATAATGAGCCAGTTCTTAA**TTCGAAACT**<br>**GGACTGCCGGACGCGCTGCCATTCGTT**AATCGCATAAGGTAATTCACAATGATTAAAGTTGAAATTAAA**TTCGTTACTGGA**<br>**CTGCCGACGCCCGTGACGAACGTT**CCATCTCAAGCCCAATTTACTACTCGTTCTGGTGTTTCTCGTCAGGGCAAGCCTTATTC<br>ACTGAATGAGCAGCTTTGTTACGTT**TTCGCTACTGGACTGTGCACAGCTCGGGCCAGCGTT**GATTTGGGTAATGAATATCC<br>GGTTCTTGTCAAGATTACTCTTGATGAAGGTCAGCCAGCCTATGCGCCTGGTCTGTA**CACCGTTCATCTGTCCTCTTTC**

Table S8. Unmodified staple sequences to fold all gBlocks™ into DNA tetrahedron.

Tetrahedron staples	Unmodified sequence
Staple-2	CAAAAGGATTATGCGATTTTAAGAACTGGAGATAAATAACGC
Staple-3	GACCAGGCGCATTTTTTAGGCTGGCTCTTTAATCATTTTTTTGTGAATTACCATTACGAGGCATTT TTTAGTAAGAGC
Staple-4	AACCCTCGTTCTGGATAGCGTCCGGTGTACAAACACTATCAT
Staple-5	TCAAGAGTAATCTCTTGAGATGGTTTAATTTCAAGACCTTCA
Staple-6	CAGAACGAGTATTTTTGTAAATTGGGTGACAAGAACCTTTTTGGATATTCATAGTGAATAAGGTT TTTCTTGCCCTGA
Staple-7	GCTCATTCTACCCAAATCAACGTAGAGGGTGCCAACAAAGCT
Staple-8	AAAACGAATTGGGAAGAAAAATCTAACACCGAGAACGTTAAT
Staple-9	CTCATTATACCTTTTTAGTCAGGACGCTAACGGAACATTTTTTACATTATTACAATACCACATTTT TTTCAACTAATGC
Staple-10	GATTTAGGAGGTAGAAAGATTCAAGCGAGAAAATTCAGTTGA
Staple-11	GGTAATAGTAATTTTTAATGTTTAGATACCAGACGACTTTTTGATAAAAACCAGGCTTTTGCATTT TTAAAGAAGTTT

Table S9. Unmodified staple strand sequences for folding the 449 nts scaffold into tetrahedron DNA-NP.

**Tetrahedron staples**	**Unmodified sequence**
Staple-1	TTAAAATTCGAGCTTTCATC
Staple-2	ACAACCCGTCCTGCCCCTGA
Staple-3	AACGTTTTTTTAATATTTTGAACATTAAATGTTTTTTGAGCGAGTAGAGTCTGGAGCTTTTTAAACAAGAGAATTGTA
Staple-4	TGGGATTTTTTAGGTTACGTGGGCGCATCGTTTTTTAACCGTGCATGGATTCTCCGTTTTTTGGGAACAAACCCGTAA
Staple-5	TGGTGCATATTCAATTAGAT
Staple-6	GGCGGAATTCAAAATATTGA
Staple-7	ATCGATGAACAAATATTTAA
Staple-8	CGGTTTTTTTGATAATCAGAAAACAGGAAGATTTTTTTGTATAAGCGGTAATCGTAATTTTTAACTAGCATGGTACCC
Staple-9	AAAGCTTTTTGCTCACCCAA
Staple-10	ATTTTTTTTTTGTTAAATCATAACCAATAGGTTTTTAACGCCATCAGCGTCTGGCCTTTTTTTCCTGTAGCCCGTTAA

Table S10. gBlocks™ sequence used to fold the tetrahedron 42-bp edge length with aptamers (Red bases represent the primer [forward] and primer binding sites [reverse], orange bases represent a stem region, the bolded black bases represent variable sequence, and blue bases represent the aptamers sequence).

**gBlocks™ sequence with one thrombin aptamer (29 nts)**
**GGACGCTATCCAGTCTAAACAT**TTTACTATTACCCCCTCTGGCAAAACTTCTTTTGCAAAAGCCTCTCGCTATTTTGGTTTTTA<br>TCGTCGTCTGGTAAACGAGGGTTATGATAGTGTTGCTCTTACTATGCCTCGTAATTCCTTTTGGCGTTATGTATCTGCATTAGTT<br>GAATGTGGTATTCCTAAATCTCAACTGATGAATCTTTCTACCTGTAATAATGTTGTTCCGTTAGTTCGTTTTATTAACGTAGATT<br>TAGTCCGTGGTAGGGCAGGTTGGGGTGACTTTCTTCCCAACGTCCTGACTGGTATAATGAGCCAGTTCTTAAAATCGCATAA<br>GGTAATTCACAATGATTAAAGTTGAAATTAAACCATCTCAAGCCCAATTTACTACTCGTTCTGGTGTTTCTCGTCAGGGCAAGC<br>CTTATTCACTGAATGAGCAGCTTTGTTACGTTGATTTGGGTAATGAATATCCGGTTCTTGTCAAGATTACTCTTGATGAAGGTC<br>AGCCAGCCTATGCGCCTGGTCTGTA**CACCGTTCATCTGTCCTCTTTC**
**gBlocks™ sequence with three-thrombin aptamer (15 nts)**
**GGACGCTATCCAGTCTAAACAT**TTTACTATTACCCCCTCTGGCAAAACTTCTTTTGCAAAAGCCTCTCGCTATTTTGGTTTTTA<br>TCGTCGTCTGGTAAACGAGGGTTATGATAGTGTTGCTCTTACTATGCCTCGTAATTCCTTTTGGCGTTATGTATCTGCATTAGTT<br>GAATGTGGTATTCCTAAATCTCAACTGATGAATCTTTCTACCTGTAATAATGTTGTTCCGTTAGTTCGTTTTATTAACGTAGATT<br>T**TT**CGATGGTTGGTGTGGTTGG**TGGAGCGCCGTCCGG**ATCG**TT**TTCTTCCCAACGTCCTGACTGGTATAATGAGCCAGTTC<br>TTAA**TT**CGAAGGTTGGTGTGGTTGG**CCGGACGCGCTGCCA**TTCG**TT**AATCGCATAAGGTAATTCACAATGATTAAAGTTG<br>AAATTAAA**TT**CGTTGGTTGGTGTGGTTGG**CCGACGCCCGTGACG**AACG**TT**CCATCTCAAGCCCAATTTACTACTCGTTCTG<br>GTGTTTCTCGTCAGGGCAAGCCTTATTCACTGAATGAGCAGCTTTGTTACGTTGATTTGGGTAATGAATATCCGGTTCTTGTCA<br>AGATTACTCTTGATGAAGGTCAGCCAGCCTATGCGCCTGGTCTGTA**CACCGTTCATCTGTCCTCTTTC**

Table S11. Design of multifunctional scaffold with alternative combinations of modifications.

Number of modification	Name of scaffold	Biotin modification of dCTPs (%)	NH_2 modification of dCTPs (%)	αThiol modification of dNTPs (%)
**2-modification**	Multi-I	-	5%	5%
	Multi-II	-	10%	10%
	Multi-III	-	20%	20%
**3-modification**	Multi-IV	5%	10%	10%
	Multi-V	7.5%	15%	15%
	Multi-VI	10%	20%	20%

Table S12. ssDNA scaffold used to fold the corresponding DNA-NPs.

DNA-NPs	Scaffolds length	Templates used
Six-Helix Bundle (6-HB)<br>Figure S1a	1,644 nts	M13mp18
Pentagonal Bipyramid (PB)<br>Figure S1b	1,616 nts	M13mp18
Tetrahedron 31 nts edge length<br>Figure S1c	449 nts	M13mp18
Tetrahedron 42 nts edge length<br>Figure S1d	522 nts	gBlocks without loop (Table S7)
Tetrahedron 42 nts edge length 1 loop<br>Figure S1d	558 nts	gBlocks with 1 loop (Table S7)
Tetrahedron 42 nts edge length 3 loops	630 nts	gBlocks with 3 loops (Table S7)
Tetrahedron 42 nts edge length 6 loops	738 nts	gBlocks with 6 loops (Table S7)
Tetrahedron 42 nts edge length 1 thrombin aptamer (29 nts long)	551 nts	gBlocks with one thrombin aptamer (Table S10)
Tetrahedron 42 nts edge length 3 thrombin aptamers (15 nts long)	648 nts	gBlocks with three thrombin aptamers (Table S10)

Table S13. Commercial enzymes evaluated for aPCR optimization of ssDNA amplification.

**Product and aPCR Reaction Composition Details**			
Enzyme Type	Taq	Taq	Taq
Enzyme Name	AccuStart Taq DNA Polymerase HiFi	OneTaq DNA Polymerase	AccuStart Long Range SuperMix
Vendor Name	Quanta Bioscience	NEB	NEB
Enzyme Abbreviation	HF	OT	AL
Provider	New England Biolabs (NEB)	NEB	Quantabio
Buffer (1x)	60 mM Tris-SO_4, 18 mM, $(NH_4)_2SO_4$, 2 mM MgSO4, pH 8.9	20 mM Tris-HCl<br>22 mM NH4Cl<br>22 mM KCl, 1.8 mM MgCl2, 0.06% IGEPAL® CA-630, 0.05% Tween® 20, pH 8.9	N/A
Forward Primer (5')	1 uM	1 uM	1 uM
Reverse Primer (3')	20 nM	20 nM	20 nM
dNTP	200 uM	200 uM	N/A (included in Buffer)
DNA Template	0.5 ug/ml	0.5 ug/ml	0.5 ug/ml
Enzyme	1.25 U	1.25 U	N/A (included in Buffer)

Table S14. Optimal aPCR thermocycler conditions for ssDNA amplification with each enzyme.

**aPCR ThermoCycler Conditions**				
Enzyme Short Name	HiFi	OneTaq	AccuStart Long	**Cycle Number**
Initial Denaturation	94C, 1 minute (min)	94C, 1 min	95C, 3 min	1
Denaturation	94C, 20 seconds (sec)	94C, 20 sec	92C, 30 sec	30-35
Annealing	55C, 30 seconds	55C, 30 sec	55C, 30 sec	
Elongation	68C, 1 min/kilobase (kb)	68C, 1 min/kb	68C, 1 min/kb	

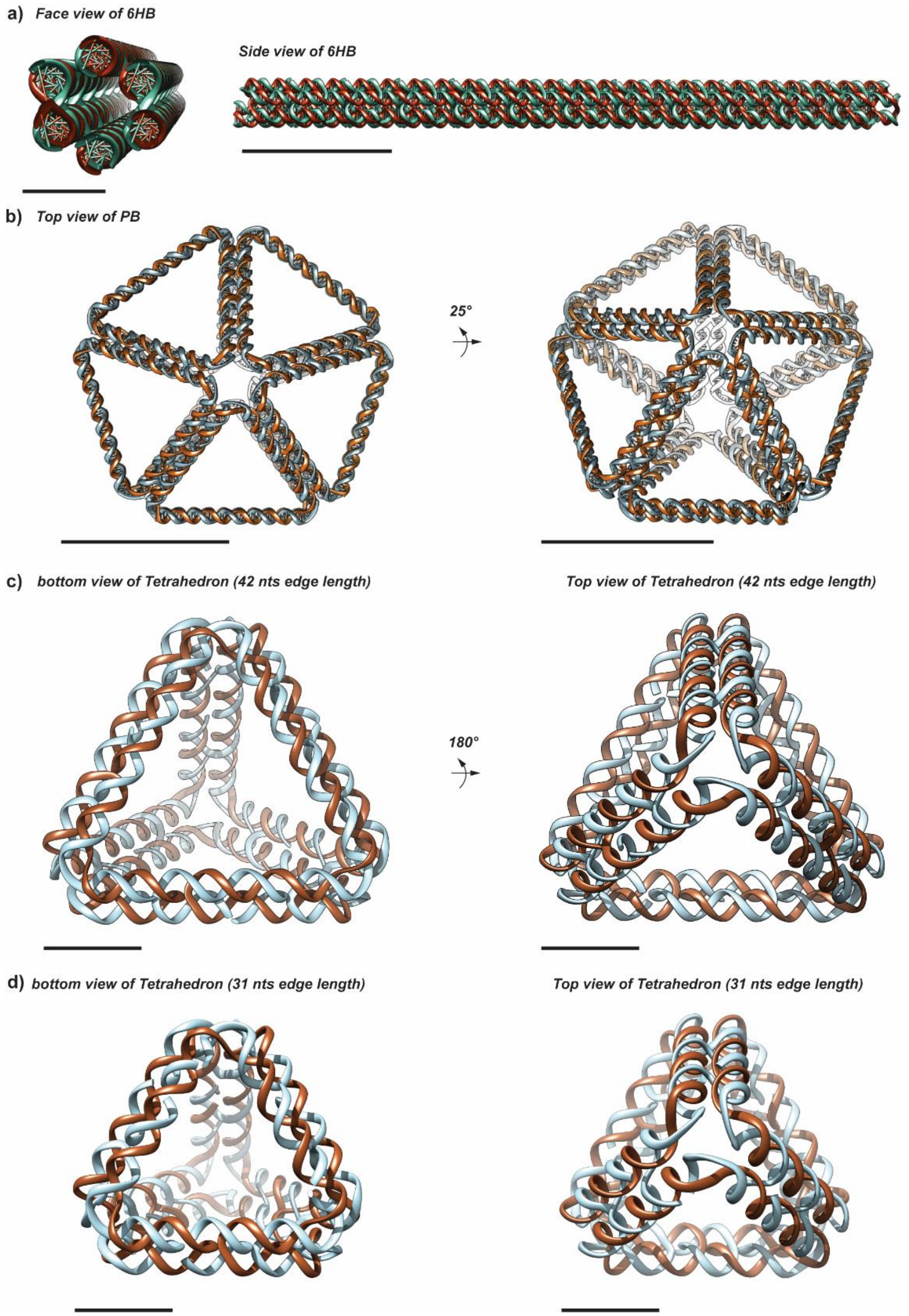

Figure S1. 3D models of the DNA-NPs (6HB and PB) folded in this study. **a)** 1D Rod-shaped DNA-NP (6HB) (Scale bars: 5 nm for the left panel and 20 nm for the right panel)**. b)** 3D PB

DNA-NP (Scale bar: 20 nm). **c)** 3D tetrahedron DNA-NP with 42 nts edge length. (Scale bar: 5 nm). **d)** 3D tetrahedron DNA-NP with 31 nts edge length. (Scale bar: 5 nm).

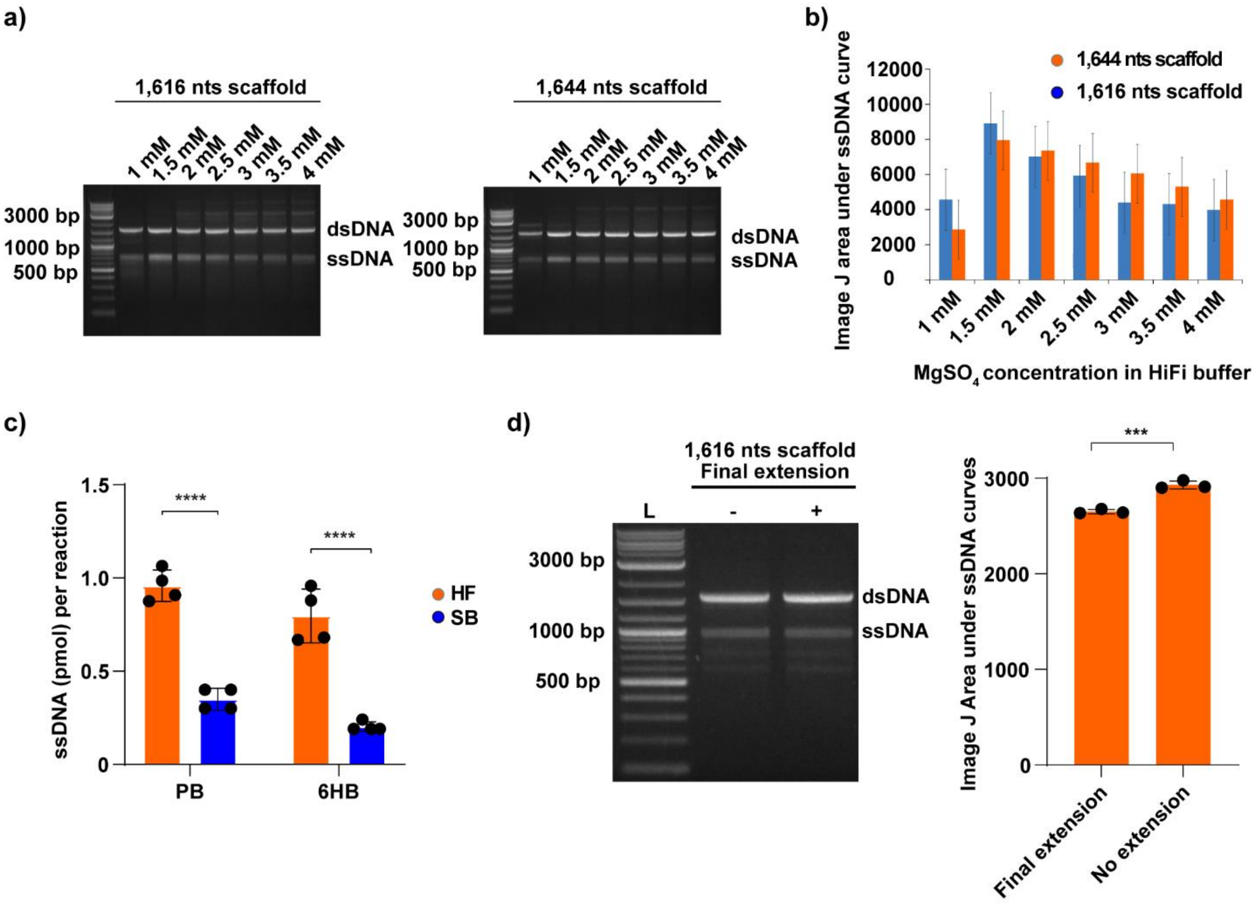

Figure S2. Optimization of aPCR reaction buffer with OT enzyme and thermocycler final extension. **a)** Gel electrophoresis showing aPCR products with different $MgSO_4$ concentrations (1-4 mM) in HiFi Buffer for the production of 1,616 nts (*left*) and 1,644 nts (*right*) ssDNA. **b)** ImageJ analysis of band brightness from gels shown in panel **(a)** as an estimate of yield by $MgSO_4$ concentration for both scaffolds. For the two highest concentrations of $MgSO_4$, 1.5 mM and 2 mM, no statistically significant difference was observed. **c)** ssDNA quantification for both scaffolds produced with OT enzyme using optimized HiFi (HF) and standard buffer (SB). **d)** Gel characterization (*left*) and ImageJ quantification (*right*) comparing ssDNA yield with and without final extension.

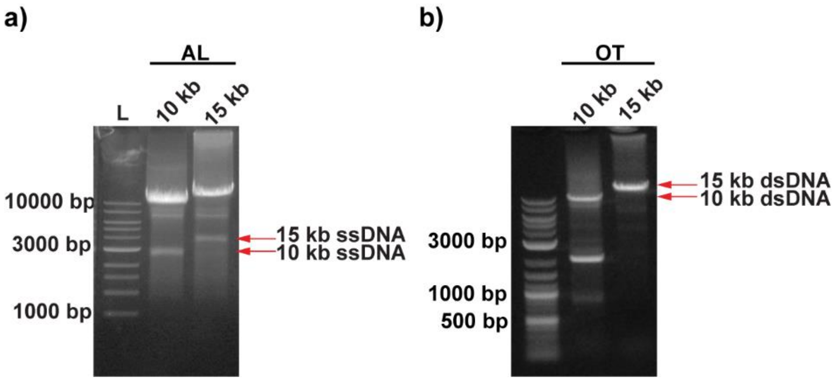

Figure S3. Production of 10 kilobases (kb) and 15 kb scaffolds with the **a)** AL and **b)** OT enzymes.

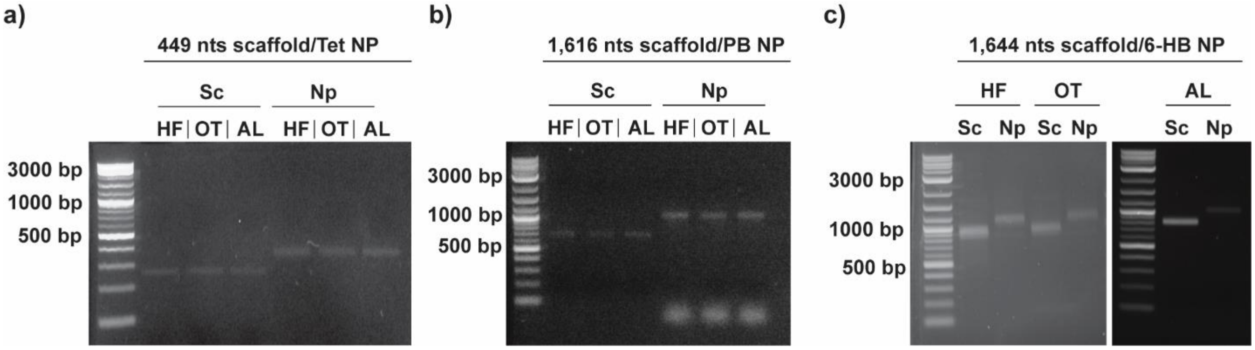

Figure S4 Gel electrophoresis showing the different scaffolds of **a)** 449 nts, **b)** 1,616 nts, and **c)** 1,644 and their corresponding folded DNA-NPs after production with the different enzymes (HiFi, HF; OneTaq, OT; AccuStart Long, AL). Gels are representative.

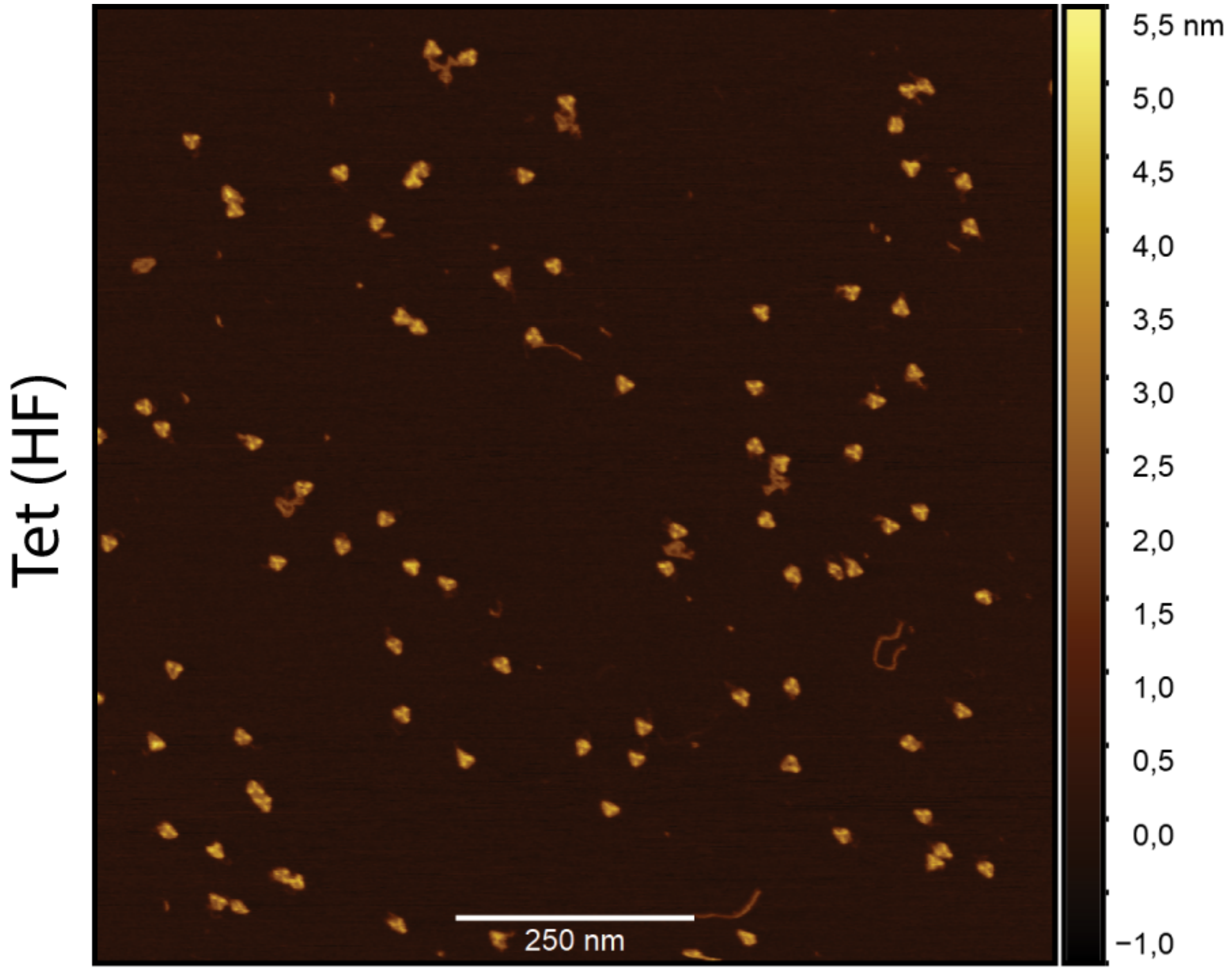

Figure S5. Full atomic force microscopy (AFM) image of tetrahedron DNA-NPs (TET) folded with the 449 nts scaffold produced with HF.

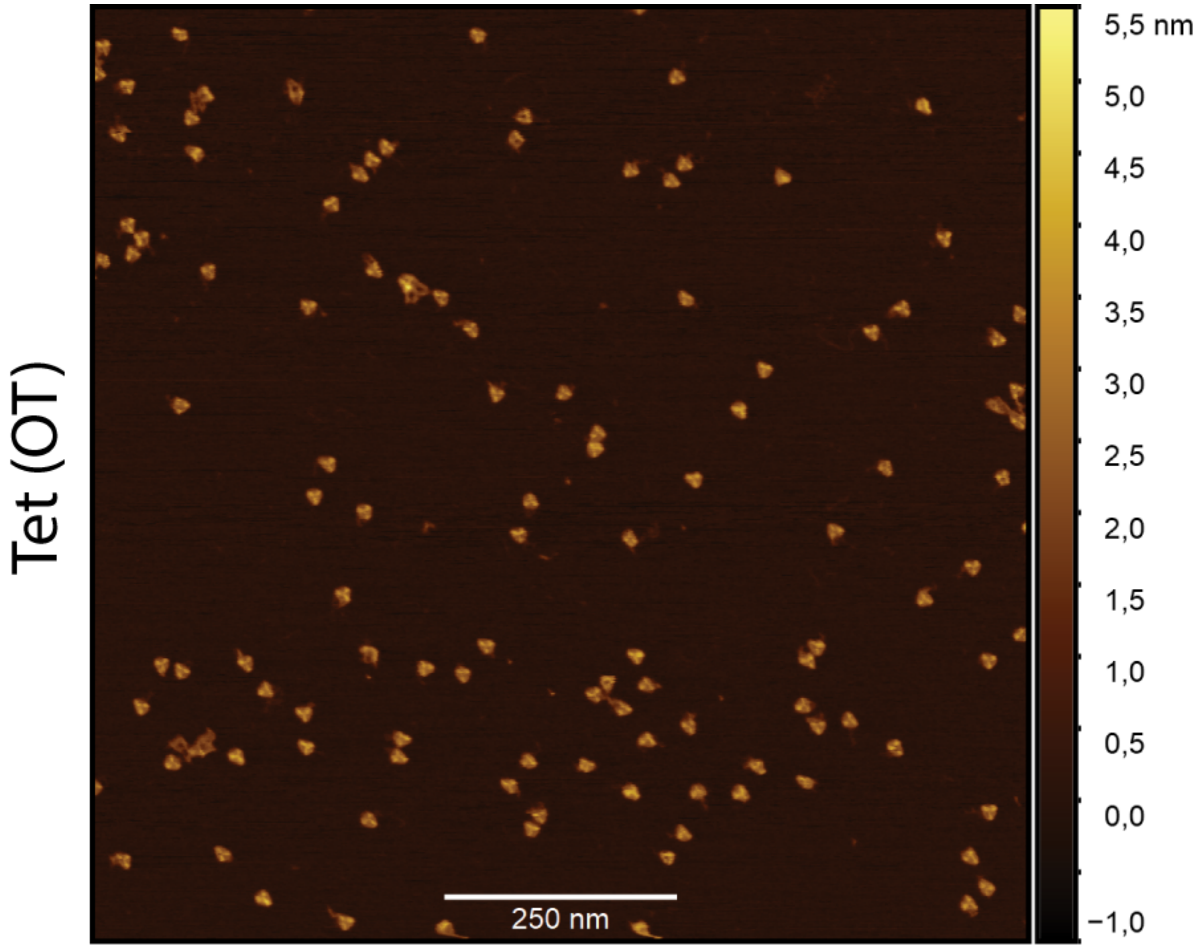

Figure S6. Full atomic force microscopy (AFM) image of tetrahedron DNA-NPs (TET) folded with the 449 nts scaffold produced with OT.

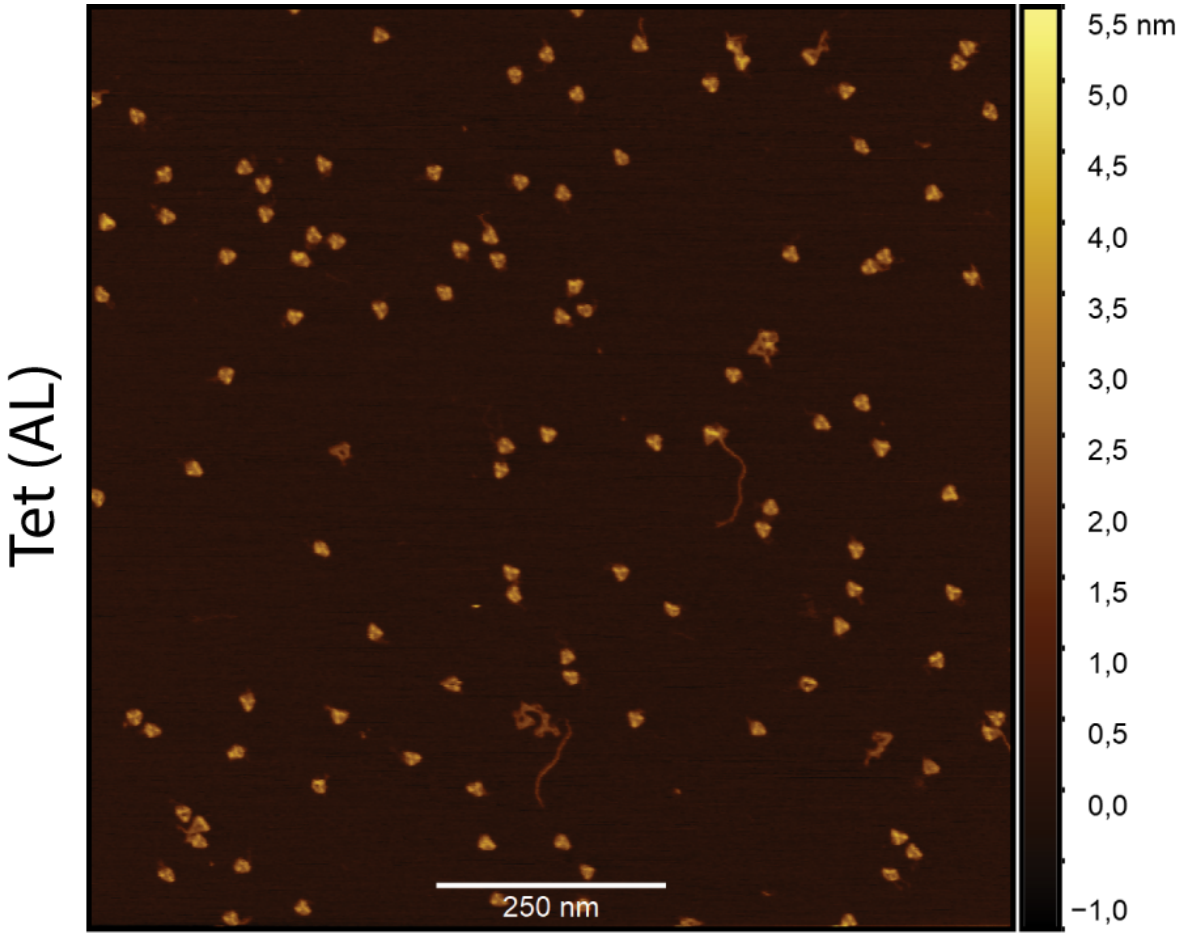

Figure S7. Full atomic force microscopy (AFM) image of tetrahedron DNA-NPs (TET) folded with the 449 nts scaffold produced with AL.

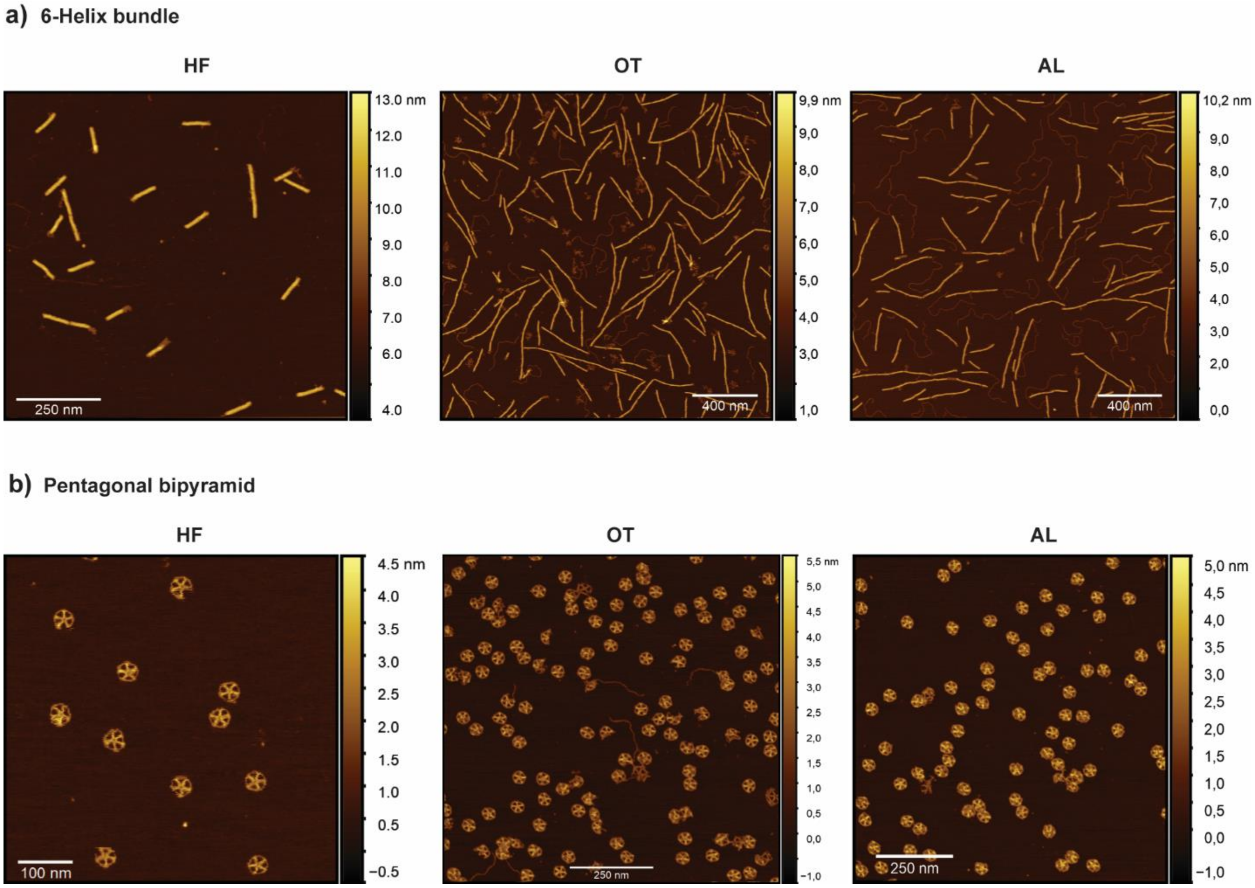

Figure S8. Full atomic force microscopy (AFM) images of **a)** 6-Helix Bundle (6-HB) and **b)** pentagonal bipyramid (PB) DNA-NPs folded with scaffolds produced with HF, OT, and AL enzymes.

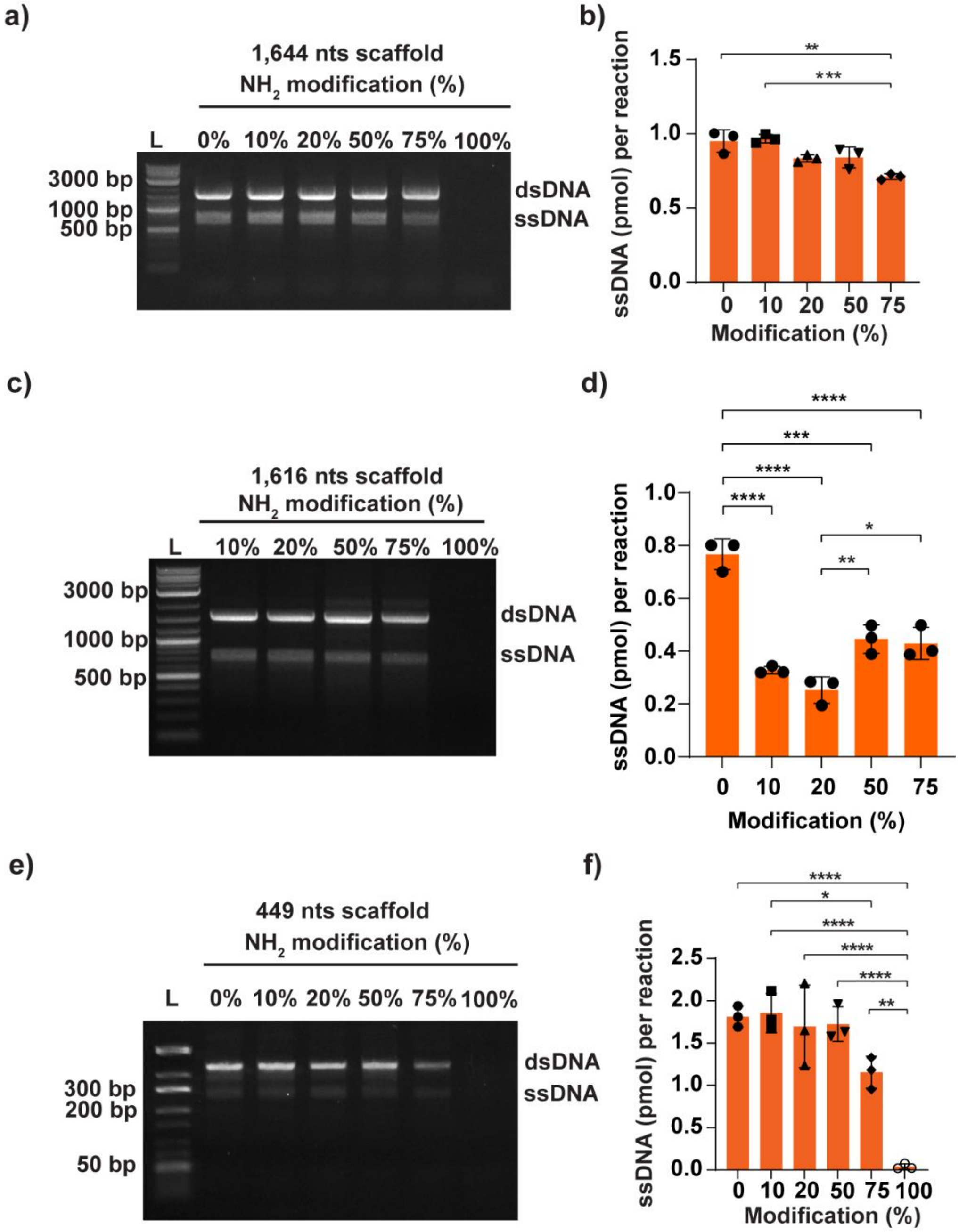

Figure S9. Synthesis and quantification of 449 nts, 1,616 nts, and 1,644 nts scaffolds with various percentages of amino (NH_2) modifications. **a)** Gel electrophoresis for 1,644 nts scaffold confirmed the presence of the aPCR products generated with the HF enzyme and different percentages of NH_2-deoxycytidine triphosphates (dCTPs) (0% to 100% NH_2-modified dCTPs). **b)** Quantity of

1,616 nts scaffold produced with various ratios NH_2-dCTPs using the HF enzyme (the 100% ratio did not yield enough ssDNA to allow purification). **c)** Gel electrophoresis for 1,616 nts scaffold confirmed the presence of the aPCR products generated with the HF enzyme and different percentages of NH_2-dCTPs (0% to 100% NH_2-modified dCTPs). **d)** Quantity of 1,616 nts scaffold produced with various ratios NH_2-dCTPs using the HF enzyme (the 100% ratio did not yield enough ssDNA to allow purification). **e)** Gel electrophoresis for 449 nts scaffold confirmed the presence of the aPCR products generated with the HF enzyme and different percentages of NH_2-dCTPs (0% to 100% NH_2-modified dCTPs). **f)** Quantity of 449 nts scaffold produced with various ratios NH_2-dCTPs using the HF enzyme (0% to 100% NH_2-modified dCTPs). Error bars represent standard deviation (SD) of the mean yields for each modified scaffold. Data were presented as mean ± SD. Statistical analyses were performed using one-way ANOVA followed by Tukey post-hoc test (n=3 samples/group, '*' $p<.05$, '**' $p<.01$, '***' $p<.001$, and '****' $p<.0001$).

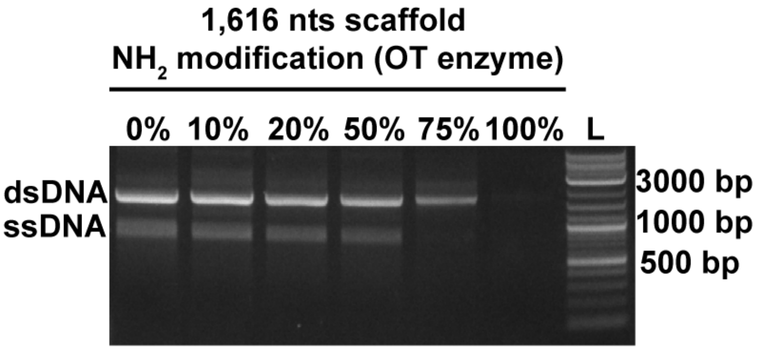

Figure S10. Gel electrophoresis of NH_2 modified scaffold produced with OT enzyme.

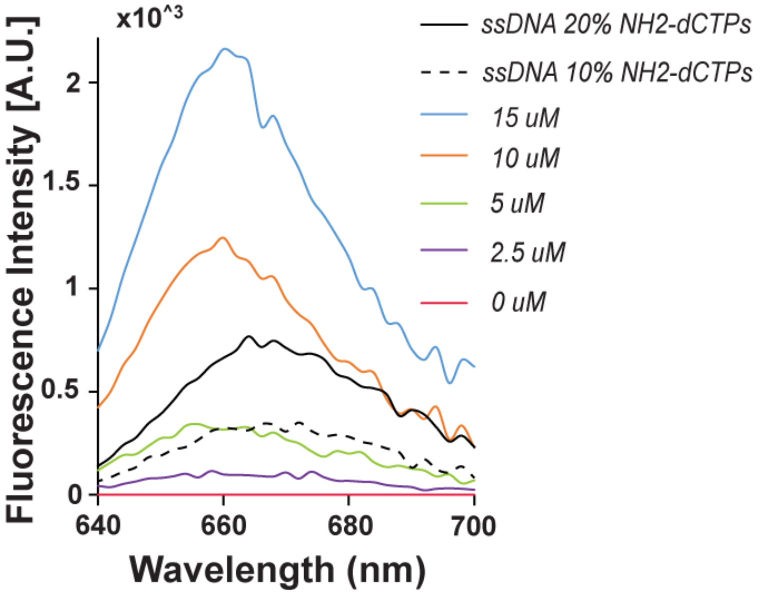

Figure S11. Cy5 modification of NH_2-modified 1,644 nts ssDNA. Emission spectra of different concentrations of free Cy5 dye (0-15 µM) were used as standard to quantify the modification of the scaffold modified with 10% and 20% NH_2-dCTPs and further conjugated with Cy5-NHS.

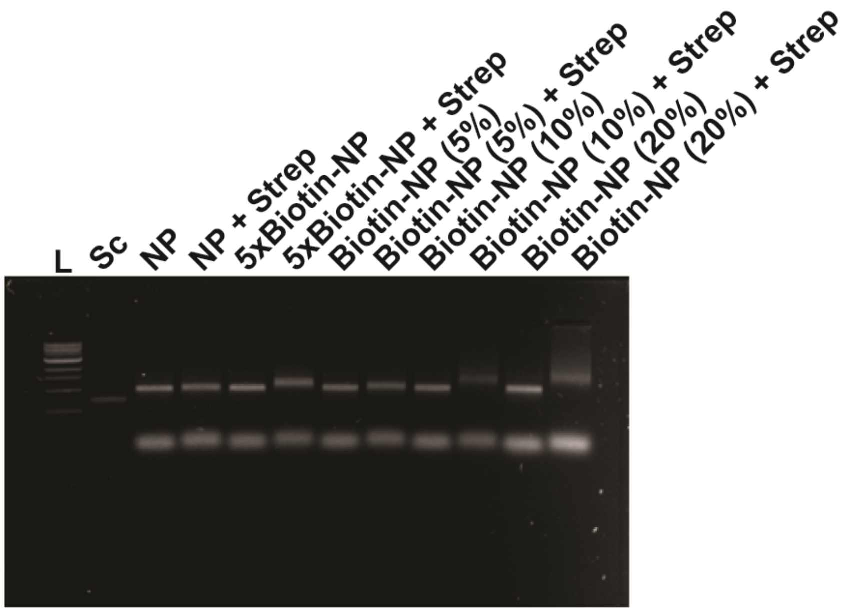

Figure S12. Detection and comparison of biotin-modifications on DNA-NPs at various percentages using streptavidin attachment. (Sc: unmodified scaffold; NP: unmodified DNA-NP; strep: streptavidin; 5xBiotin-NP: only 5-biotin containing DNA-NPs; Biotin-NP (5%): DNA-NP folded with 5% biotinylated scaffold; Biotin-NP (10%): DNA-NP folded with 10% biotinylated scaffold; Biotin-NP (20%): DNA-NP folded with 20% biotinylated scaffold).

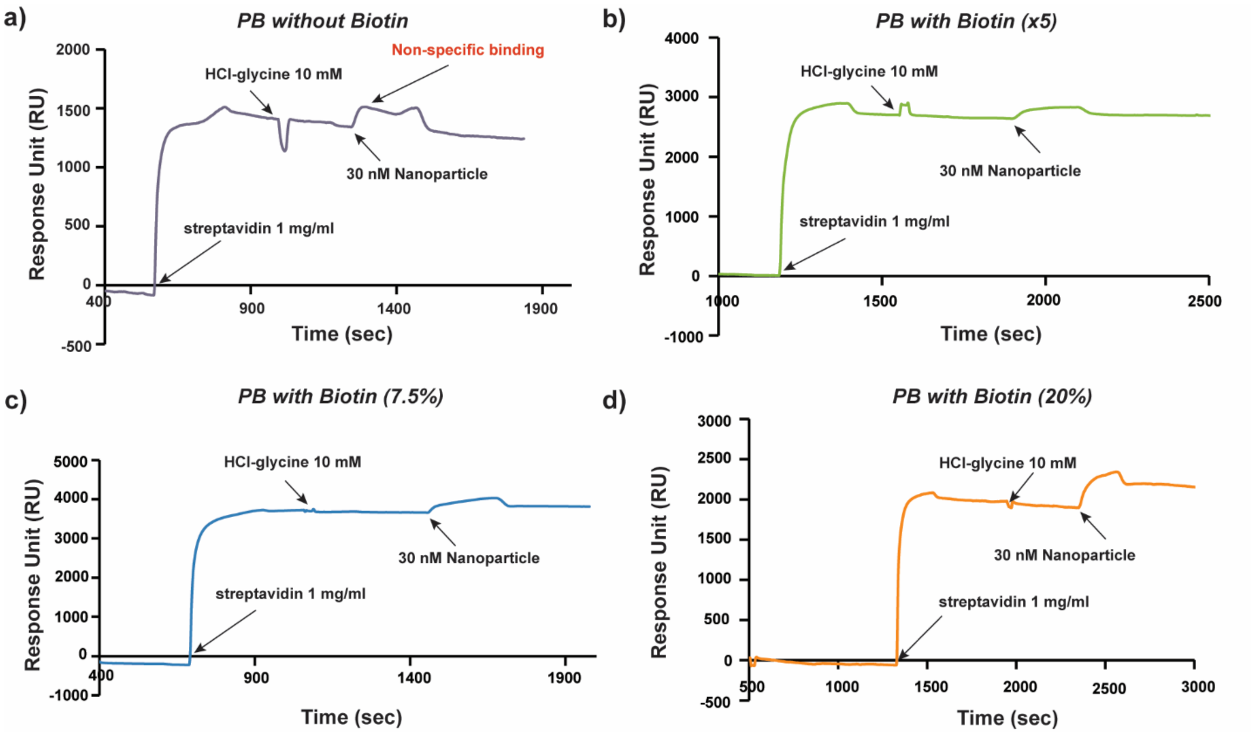

Figure S13. Representative SPR binding curves for the biotin-modified PB DNA-NPs (30 nM) on streptavidin modified surface. **a)** PB DNA-NP without modification used as a negative control. **b)** Binding of DNA-NPs folded with biotinylated staple strands (5 biotin group). **c)** DNA-NPs folded with scaffold synthesized with 7.5% biotin-dCTPs. **d)** DNA-NPs folded with scaffold synthesized with 20% biotin-dCTPs.

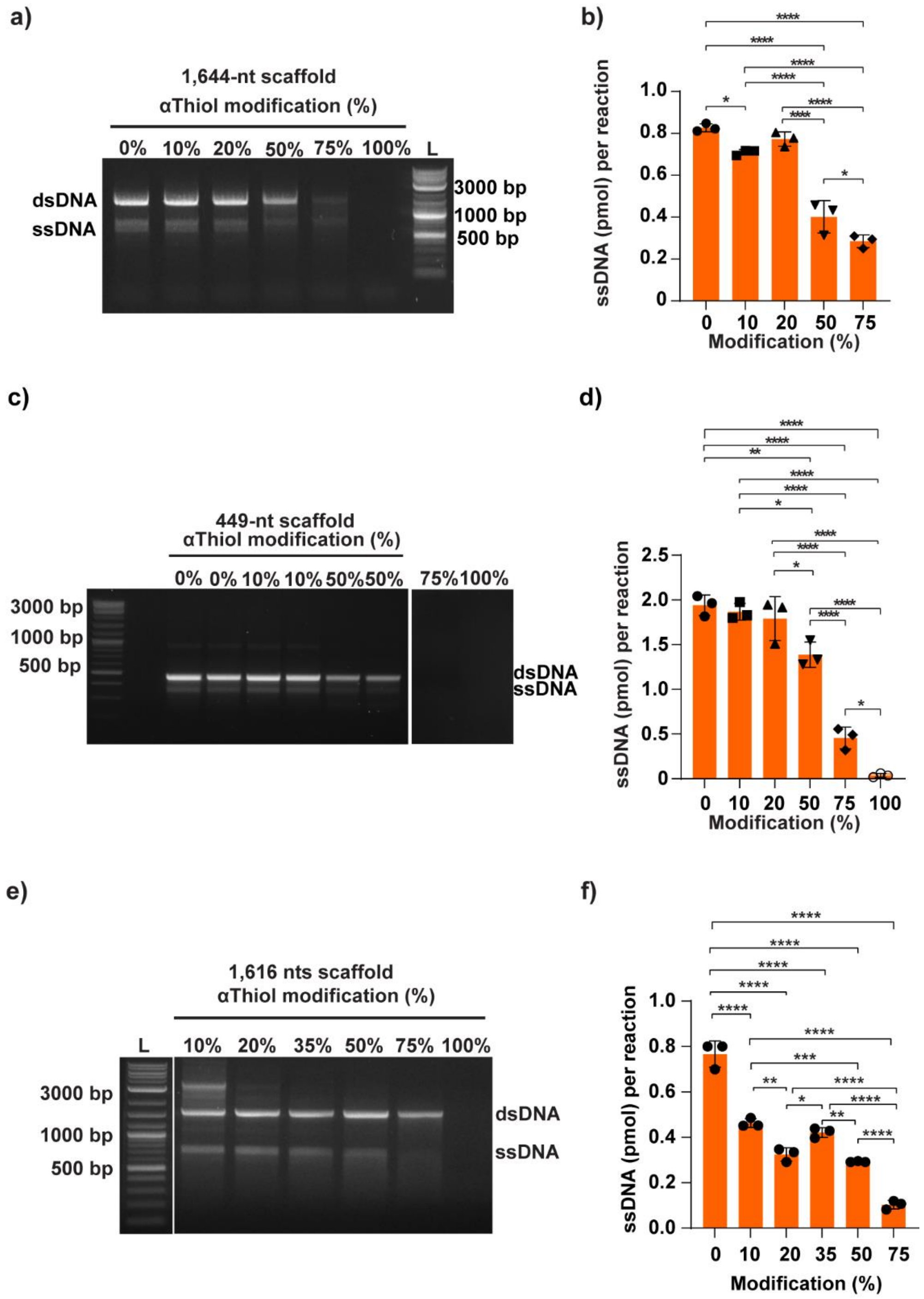

Figure S14. Synthesis and quantification of 449 nts, 1,616 nts, and 1,644 nts scaffolds modified with various percentages of αThiol modifications. **a)** The efficiency of the 1,644 nts scaffold synthesis in the presence of HF enzyme and different percentages (0% to 100%) of αThiol dNTPs was compared in the agarose gel electrophoresis according to the band intensities. **b)** Data from

1,644 nts scaffold (with 0% to 75% of αThiol-modified dNTPs) synthesized as triplicate were represented in the bar graph. **c)** The efficiency of the 449 nts scaffold synthesis in the presence of HF enzyme and different percentages (0% to 100%) of αThiol dNTPs was compared in the agarose gel electrophoresis according to the band intensities. **d)** Data from each scaffold (with 0% to 100% of αThiol-modified dNTPs) synthesized as triplicate were represented in the bar graph. **e)** The efficiency of the 1,616 nts scaffold synthesis in the presence of HF enzyme and different percentages (0% to 100%) of αThiol dNTPs was compared in the agarose gel electrophoresis according to the band intensities. **f)** Data from each scaffold (with 0% to 75% of αThiol-modified dNTPs) synthesized as triplicate were represented in the bar graph. Data obtained from each modified scaffold were shown as mean ± SD in the bar graph and analyzed using one-way ANOVA and Tukey post-hoc test (n=3 samples/group, '*' $p<.05$, '**' $p<.01$, '***' $p<.001$, and '****' $p<.0001$).

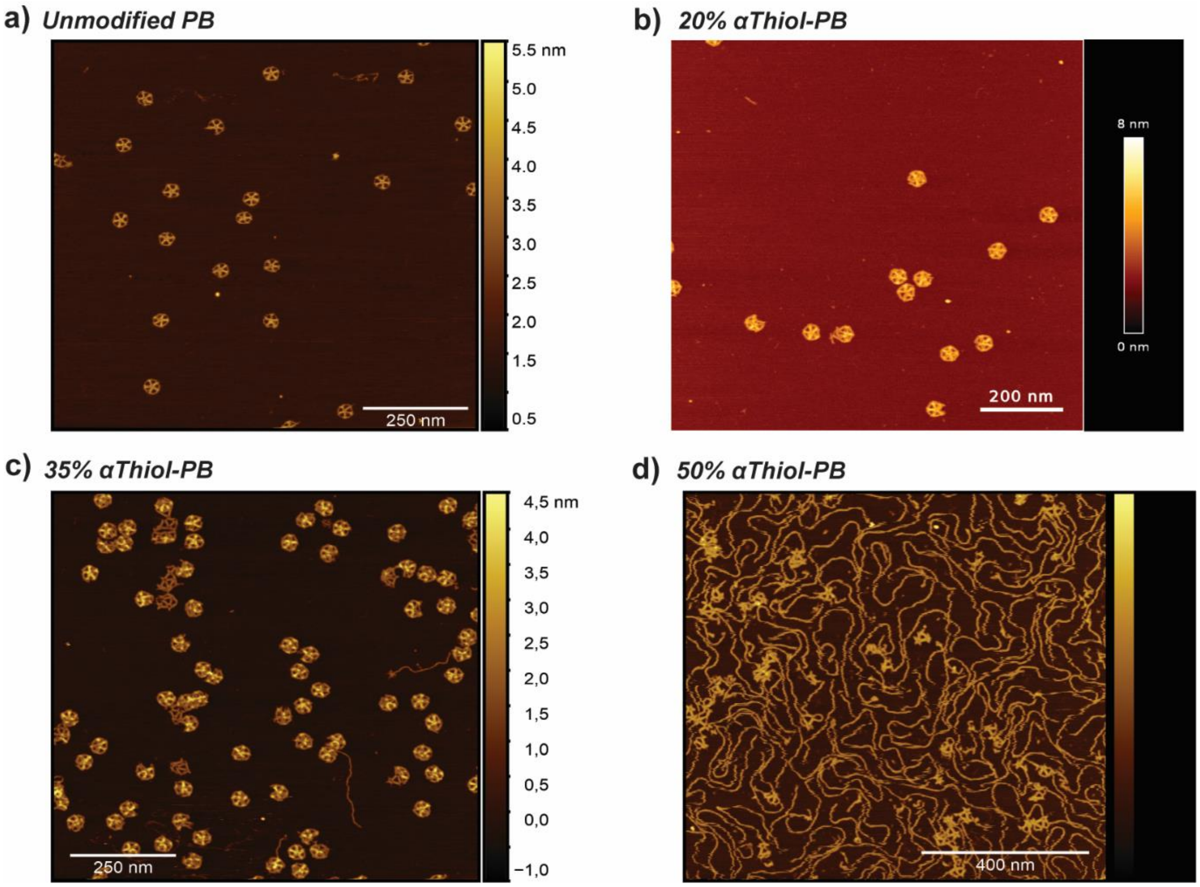

Figure S15. AFM images of PB DNA-NPs incubated in 20% mouse serum. **a)** PB DNA-NP folded with unmodified scaffold using unmodified staple strands, **b)** PB DNA-NP folded with 20% αThiol-modified scaffold using unmodified staple strands, **c)** PB DNA-NP folded with 35% αThiol-modified scaffold using unmodified staple strands, and **d)** PB DNA-NP folded with 50% αThiol-modified scaffold using unmodified staple strands.

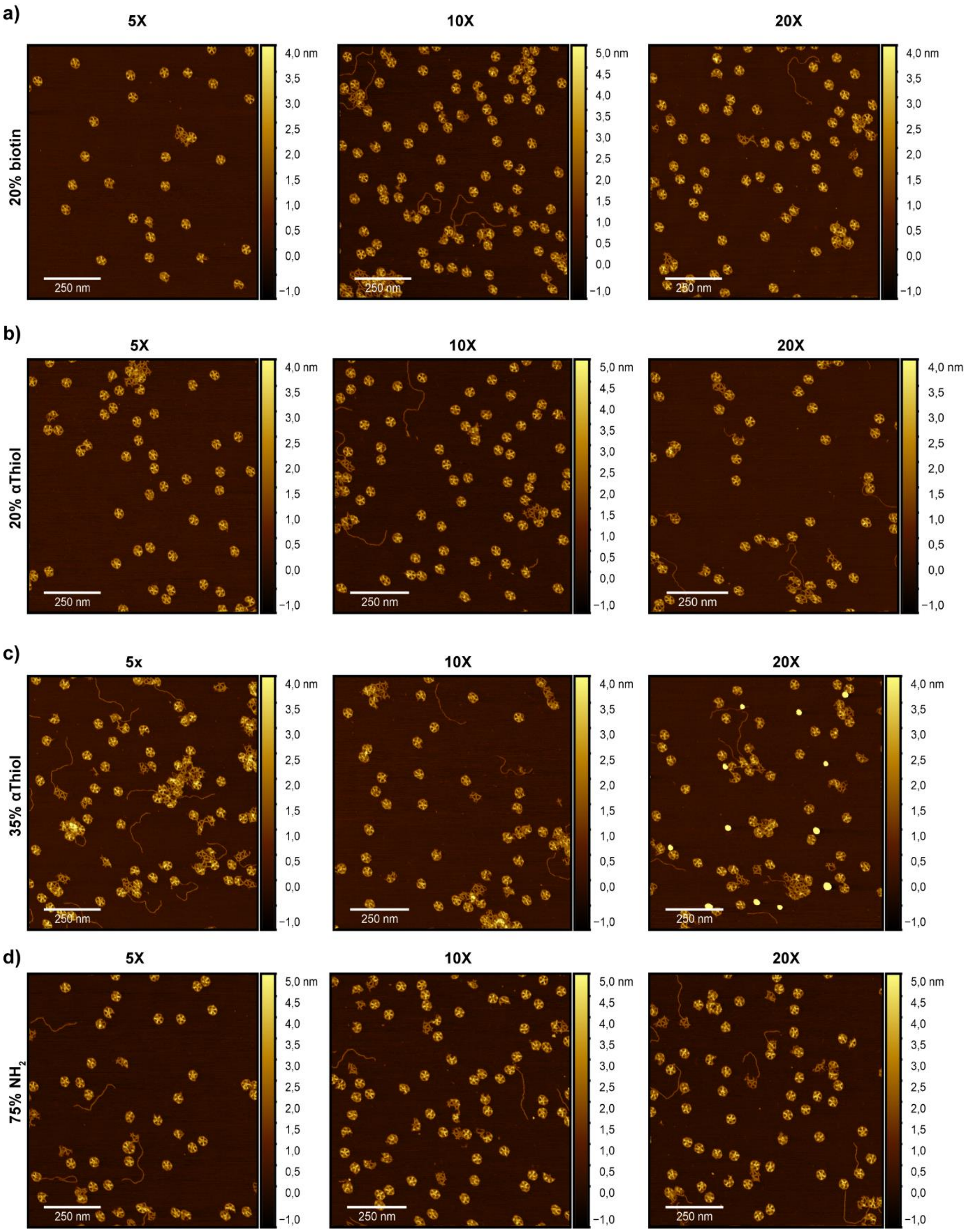
a)
5X
10X
20X
20% biotin
b)
20% αThiol
c)
5x
35% αThiol
d)
75% NH2
250 nm
4,0 nm
5,0 nm
3,5
3,0
2,5
2,0
1,5
1,0
0,5
0,0
−1,0

Figure S16. Full AFM images of DNA NPs folded with four different modified scaffolds in presence of three different molar concentrations of staple mix (5X, 10X, and 20X molar ratios). **a)** Scaffold produced with 20% biotin-dCTPs, **b)** scaffold produced with 20% αThiol-dNTPs, **c)** scaffold produced with 35% αThiol-dNTPs, and **d)** Scaffold produced with 75% Amino-dCTPs.

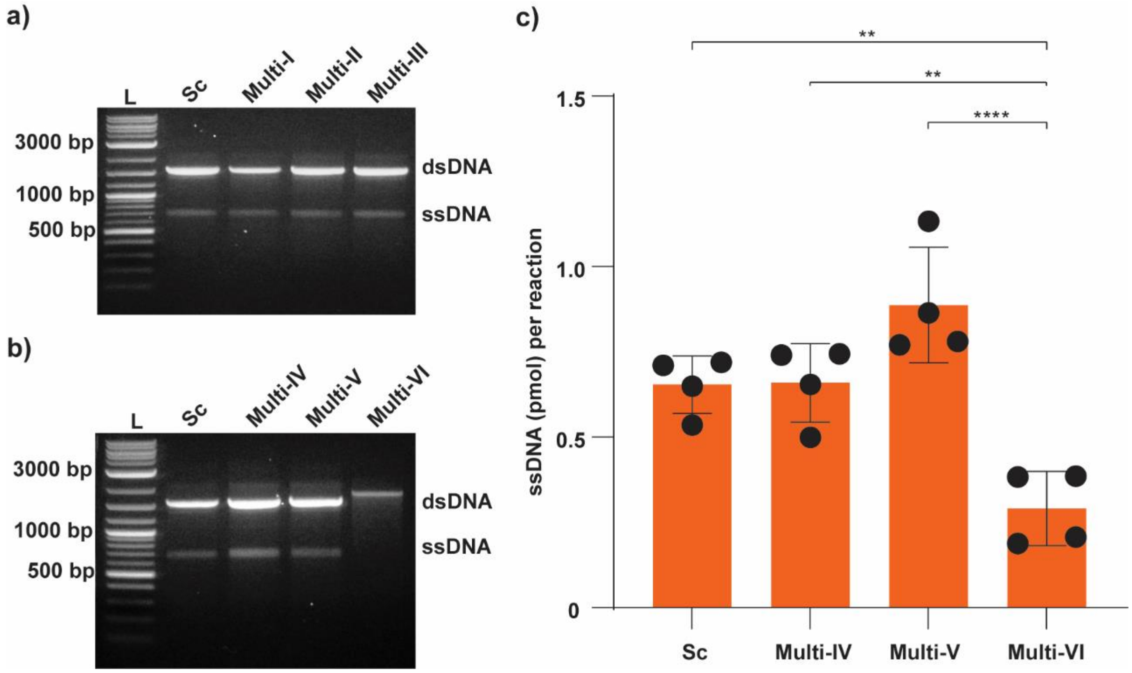

Figure S17. Production and quantification of multifunctional 1,616 nts length scaffold. **a)** Gel electrophoresis for the scaffolds with two modifications. **b)** Gel electrophoresis for the scaffolds with three modifications. **c)** Quantity of scaffolds (pmol per 50 µl reaction) were shown as mean ± SD in the graph and analyzed using one-way ANOVA and Tukey post-hoc test (n=4 for each sample; '**' p<.01, and '****' p<.0001).

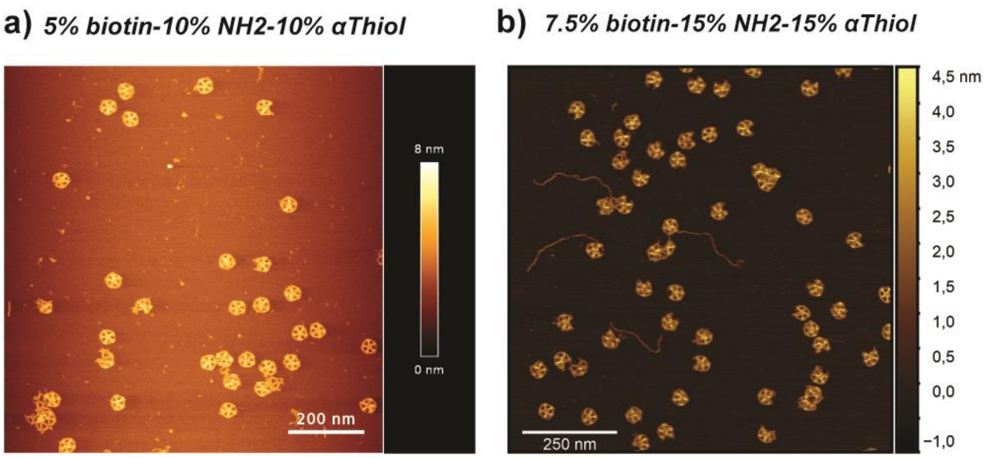

Figure S18. Full AFM images of **a)** PB DNA-NPs folded with 5% biotin-10% NH_2-10% αThiol- (Multi-IV) and **b)** 7.5% biotin-10% NH_2-10% αThiol- (Multi-V) modified scaffolds.

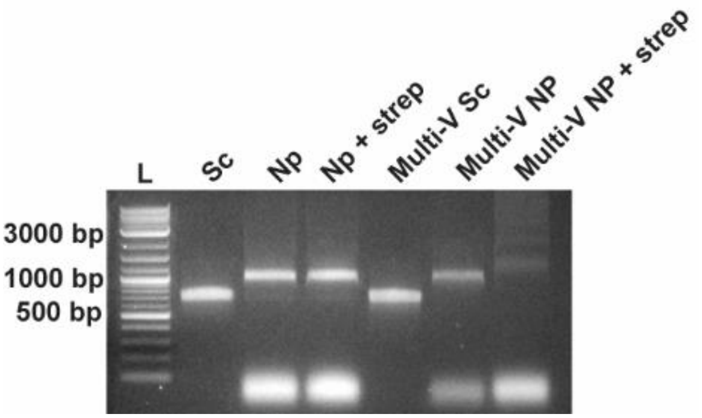

Figure S19. Detection of streptavidin binding to the DNA-NPs with biotin modification. Sc: unmodified scaffold; NP: unmodified DNA-NP; strep: streptavidin; Multi-Sc: multifunctional scaffold with 7.5% biotin-15% NH_2-15% aThiol; Multi-V NP: multifunctional DNA-NP folded with multifunctional scaffold with 7.5% biotin-15% NH_2-15% aThiol. The red arrow indicates the streptavidin-bound multi-NP.

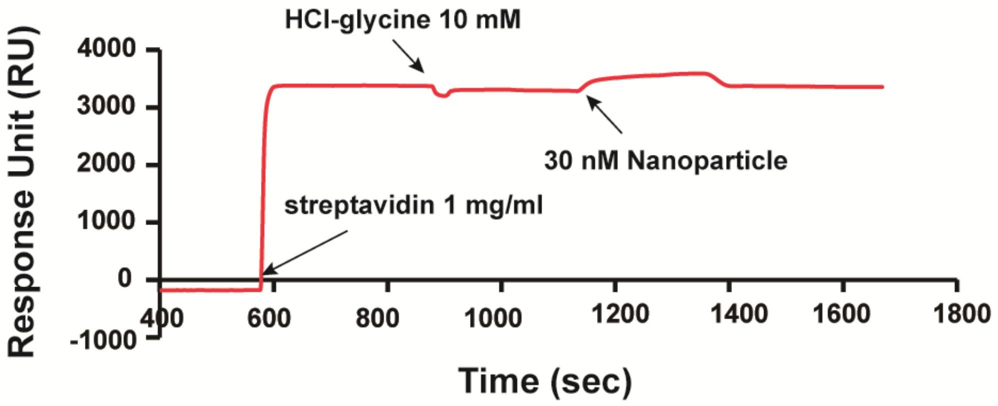

Figure S20. Representative SPR binding curve of the Multi-V DNA-NP folded with scaffold produced with 7.5% biotin-dCTPs.

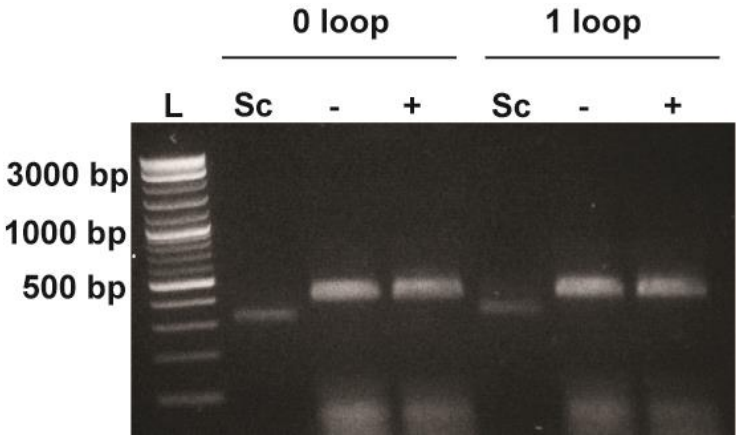

Figure S21. Gel characterization of DNA tetrahedron NPs containing 0 and 1 loop. Sc: scaffold; '-' denoted no incubation with ProteinG-PNA; '+' denoted incubation with ProteinG-PNA.

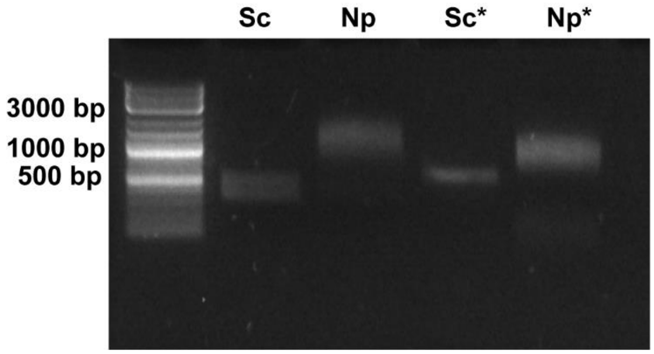

Figure S22. Comparison of gBlocks-derived DNA-NPs folded with either modified scaffold or modified staples. Particularly, in this gel, Sc indicates the DNA scaffold amplified from the gBlocks and Np indicates the NP folded with modified staples. Sc* indicates DNA scaffold with 6-loop integrated amplified from gBlocks and Np* denotes for the NP folded with the 6-loops modified scaffold.

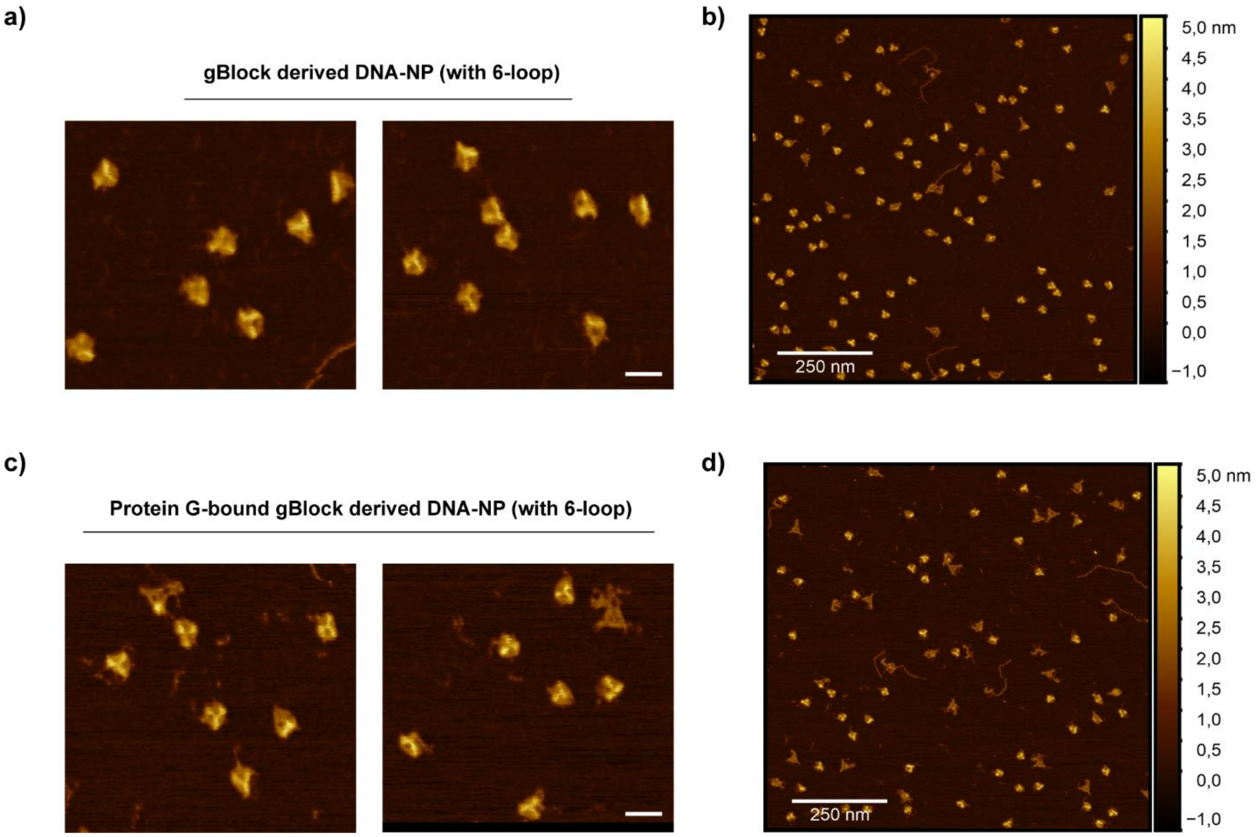

Figure S23. Full AFM images of DNA-NPs. **a)** Representative image of Bare gBlock-derived DNA-NPs (folded with 6-loop integrated scaffold) (scale bar: 50 nm) and **b)** its original image from AFM. **c)** Representative image of protein G (PG)-functionalized gBlock-derived DNA-NP (folded with 6-loop integrated scaffold) (scale bar: 50 nm) and **d)** its original image from AFM.

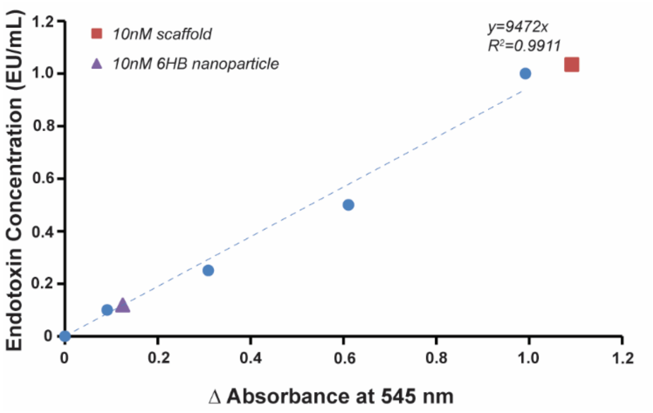

Figure S24. Representative graph of the endotoxin assay quantification performed with synthetized scaffolds (1,644 nts) and a sample of folded DNA-NP (6HB). The red square for 10 nM 1,644 nt scaffold has an endotoxin level of 1.03 EU/ml and the purple triangle for the 10 nM 6HB DNA-NP has an endotoxin level of 0.12 EU/ml.
